# The Design, Development, and Usability Testing of an eHealth Program for Youths With Osteogenesis Imperfecta: Protocol for a 2-Phase User-Centered Mixed Methods Study

**DOI:** 10.2196/47524

**Published:** 2023-06-23

**Authors:** Argerie Tsimicalis, Jennifer Stinson, Kelly Thorstad, Frank Rauch, Reggie Hamdy, Khadidja Chougui, Sofia Addab, Telma Palomo, Mitchell Bernstein, Noemi Dahan-Oliel, Louis-Nicolas Veilleux, Laura Massochin Nunes Pinto, Raissa Passos dos Santos

**Affiliations:** 1 Shriners Hospitals for Children-Canada Montreal, QC Canada; 2 The Hospital for Sick Children Toronto, ON Canada; 3 Hospital Sepaco São Paulo Brazil; 4 McGill University Montreal, QC Canada

**Keywords:** eHealth program, osteogenesis imperfecta, self-management, youth, transition of care

## Abstract

**Background:**

Innovative approaches are needed to address the self-management needs of youths with osteogenesis imperfecta (OI) transitioning into adult-oriented health care systems. Using a sequentially phased research approach, the goal is to design, develop, and test the usability of an innovative eHealth program called “Teens Taking Charge: Managing OI Online,” hereafter named “Teens OI.” This program seeks to optimize self-management, facilitate a successful transition to adult care, and address a critical gap in the quality of care for youths with OI.

**Objective:**

The study objectives are to (1) design and develop an English and French version of the Teens OI and (2) test the usability of the Teens OI in terms of efficiency, effectiveness, and satisfaction from the perspectives of youths with OI and their parents.

**Methods:**

A user-centered design is presently in progress to design and develop Teens OI. A “Website Design and Development Council” (ie, Council) has been convened, with 20 youths and parent dyads recruited and global experts surveyed at an international meeting. With unanimous support from the Council, usability testing of the Teens OI will ensue in 4 iterative cycles with 32 youth-parent dyads. All sociodemographic and usability metrics will be descriptively analyzed. All recorded interview and focus group data are analyzed using content analysis techniques involving an iterative process of data reduction, data display, conclusion drawing, and verification.

**Results:**

As of December 2022, an 8-person, interdisciplinary Teens OI council, comprising 4 health care professionals, 3 youths and young adults with OI, and 1 parent, has been convened to oversee the design and development of Teens OI. Two cycles of interviews have been conducted with 10 youths with OI with or without their parents (n=6) from December 2021 to September 2022. Data analysis has been in progress since April 2022. Aim 2 is ethically approved and will commence following the completion of content development, expected by late July 2023. Preliminary analysis indicates that the following topics need to be prioritized for the youths: mental health, pain, accessibility, medical care, education, community, and parental care.

**Conclusions:**

The proposed study will design and develop a self-management and transitional care program for youths with OI in partnership with patients, caregivers, and health care professionals. This study leverages youths’ openness to adopt eHealth technologies to meet their needs and has the potential to actively engage them to autonomously manage their lifelong conditions, and facilitate a successful transition to adult health care. Finally, the proposed study will also address a critical gap in the quality of care and the growing concern that the OI population transitioning from pediatric to adult care is at risk of various adverse events associated with the transition.

**International Registered Report Identifier (IRRID):**

DERR1-10.2196/47524

## Introduction

### Background

There is a critical demand for effective self-management and transitional care programs to optimize the quality of care and quality of life of youths and young adults with rare diseases worldwide. Transition is “the purposeful, planned movement of adolescents and young adults with chronic physical and medical conditions from child-centered to adult-oriented health care systems” [1]. Barriers impede a successful transition [2], leading to adverse events for the transitioning youths [3-8]. eHealth offers an innovative approach to address the self-management needs of transitioning youths [9,10]. Self-management is “the interaction of health behaviors and related processes that patients and families engage in to care for a chronic condition” [11]. In a burgeoning field, self-management interventions, delivered over the internet, can improve selected outcomes in certain childhood illnesses [9]. One promising program is Teens Taking Charge. Our team aims to develop this program for youths with osteogenesis imperfecta (OI). Our preliminary work showcases the critical need and motivation of youths with OI, their parents, and clinicians to engage in such a program [12-18] while addressing the global gap in OI self-management and transitional care programs [12,14,16-19]. Priority topics include a description of OI [16]; self-management of symptoms such as pain [20-22] and constipation [23]; coping with isolation, feeling different, and fear of fractures [24]; need for social support [24,25]; preparing for the transfer and navigation of the adult health care system [16-18]; managing costs [26]; and understanding the clinical manifestations of OI in adulthood [27].

“Teens Taking Charge” is a promising self-management and transitional care program available for youths with OI. Stinson et al [28] designed and developed “Teens Taking Charge,” which is currently available for youths with juvenile idiopathic arthritis (JIA), cancer, hemophilia, or organ transplants. These multilayer programs contain self-guided, interactive, multicomponent modules that consist of disease-specific education, self-management strategies, and social support. To help encourage healthy behavior among youths, 2 modules specifically target parents. Recent findings from the 10-site, randomized controlled trial in the United States, using an “active” control group (n=144), supported a modest benefit for pain and quality of life for the Teens JIA group (n=145) [29]. The program is now publicly available and being adapted for use in Ireland [30]. Pilot randomized control trial studies suggest design feasibility, program benefits, and significant improvements in disease-specific knowledge (*P*=.004), self-efficacy (*P*=.007), and transition preparedness (*P*=.046) than the control group (n=13) [31-33]. The pilot testing of “Teens Cancer” is presently in progress.

To our knowledge, there are currently no eHealth interventions designed for youths with OI and their parents, resulting in unmet needs. The typical clinical appearance of youths with OI, a rare genetic disorder (1:10,000), is bone fragility [34]. In North America, the largest cohort of pediatric patients with OI (n=400) is followed at Shriners Hospitals for Children-Canada (SHC-Canada; ie, the study site of this protocol). The health care team is internationally acclaimed for establishing the worldwide standard of care, pioneering the treatment, and testing its effectiveness. Despite these accolades, ongoing multistakeholder service evaluations conducted at the study site have identified a critical need to develop a self-management and transitional care program delivered over the internet [12,14,16-18].

### Study Goals and Aims

**Figure 1:**
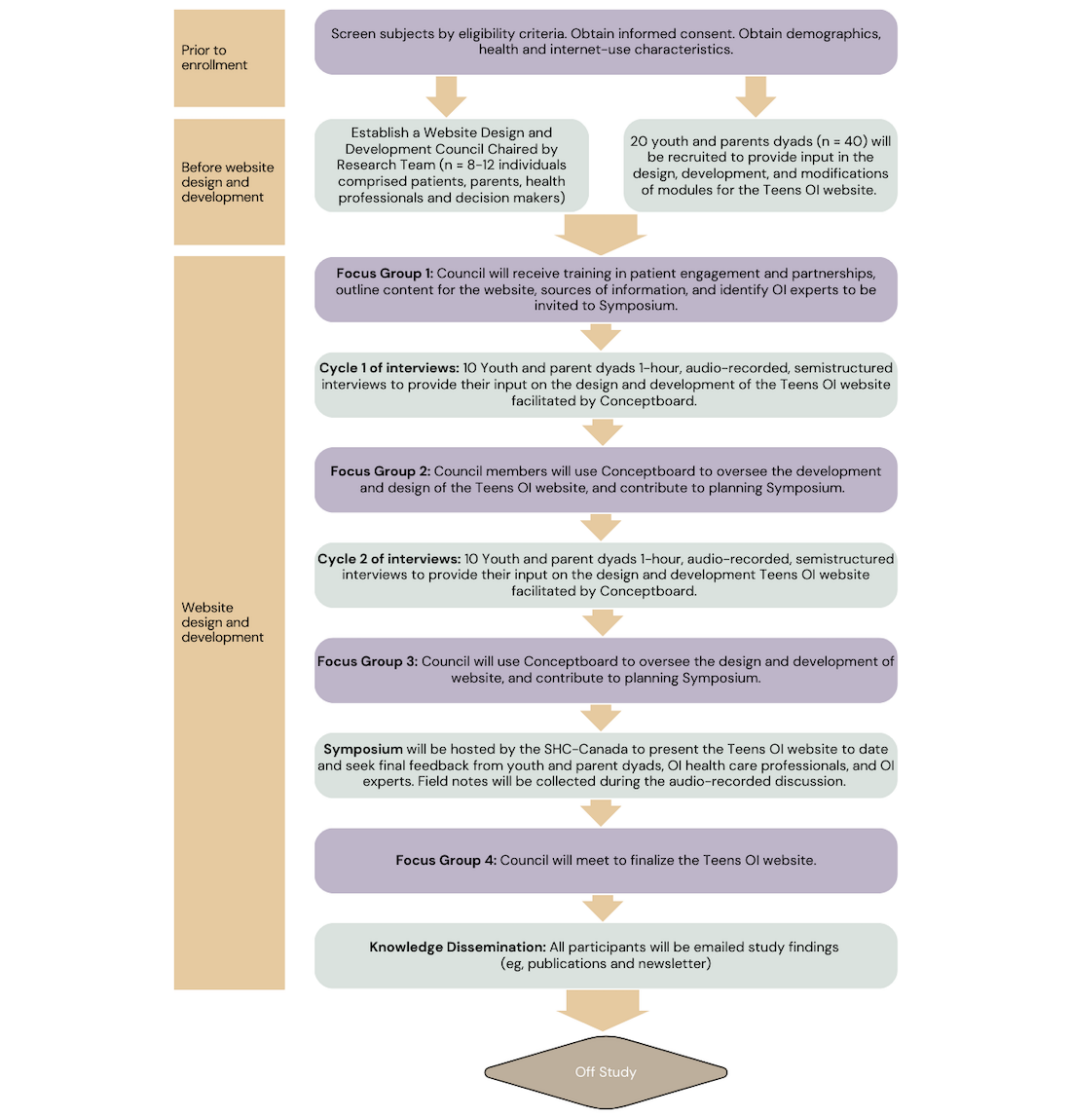
Study schema: Teens OI Phase I. OI: osteogenesis imperfecta.

**Figure 2:**
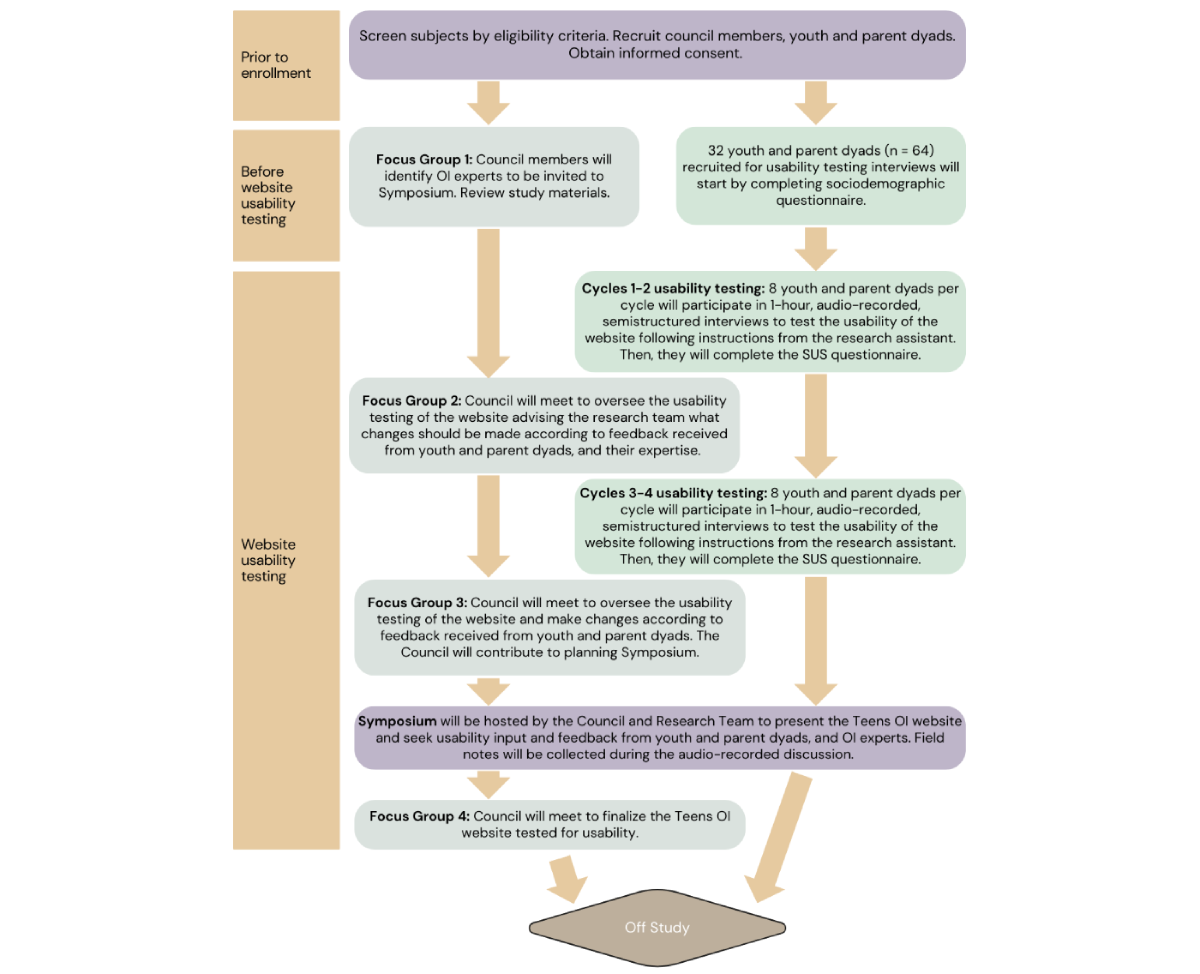
Study schema: Teens OI Phase II. OI: osteogenesis imperfecta; SUS: System Usability Scale.

With multistakeholder input from patients, families, clinicians, and the greater OI community, our research team seeks to design, develop, and test the usability of Teens Taking Charge: Managing OI Online (Teens OI)*.* This study protocol incorporates the preliminary work led by the research team. The approach is modeled after similar and successful approaches conducted with youths with JIA [29,30,32,35], hemophilia [31,36,37], cancer [38,39], and solid transplant [33,40,41]. A sequential phased approach of (1) needs assessments [14,16] (completed), knowledge syntheses [17,18,24,25,27,42,43] (completed), and preliminary symptom assessments [22,23] (completed); (2) program design, development, and usability testing [28,31,41] (this protocol), and (3) feasibility testing and outcome evaluation [32] (future research) has been adopted. The program design, development, and usability testing have 2 study aims. Aim 1 consists of designing and developing an English and French version of the Teens OI program (Figure 1). Aim 2 entails testing the usability of Teens OI in terms of (1) efficiency, (2) effectiveness, and (3) satisfaction, including perceived ease of use (ie, error prevention, other outcomes, information needs, and memorability), and perceived usefulness (ie, learnability, competency, performance speed, flexibility, and customizability) from the perspective of youths with OI and their parents (Figure 2).

### Preliminary Work for Aims 1 and 2

The preliminary work conducted for aim 1 consisted of needs assessments, knowledge syntheses, and the hiring of a former patient to inform the development of Teens OI. A usage-focused evaluation of the OI transitional care program at the study site revealed the need for a web-based self-management program [14]. Efforts to advance this mandate by the study site included the hiring of Chougui, a former patient and young adult with OI who is now a doctoral student in psychology, as a research team member since 2015. Chougui has contributed to drafting the preliminary content of Teens OI and creating varying transition-related tools [17,18,20,25-27,42]. The drafts have been informed by our completed study on self-management [16] and varying knowledge syntheses [17,18,24,25,27,42,43] and preliminary symptom assessments [22,23].

The preliminary work conducted for aim 2 has consisted of 4 qualitative usability studies conducted with 56 English-speaking and 26 French-speaking youths with JIA (n=19) [28], cancer (n=24) [38], hemophilia (n=18) [31], or transplant (n=21) [41], and one of their respective parents, to determine the usability of the user interface and content areas and refine the prototype accordingly using 2-4 iterative cycles. Youths and parents provided similar as well as differing suggestions on how to improve the website’s user interface and usability. Participants reported suggestions for minor prototype changes across all 4 programs. They also responded positively to the website’s content, use, appearance, and theme; indicated that the program was easy to navigate and understand; and perceived the program as helpful and acceptable. Many participants responded that the interactive features made them feel supported and “not alone” in their illness and that they felt enriched by their experience. No major differences between English- and French-speaking participants were found. Usability themes that arose from the 4 studies were: (1) aesthetics, (2) content and features, (3) functionality, (4) understandability, (5) comprehensiveness, (6) quality and credibility, (7) sociability, (8) intent for future use, (9) user satisfaction, and (10) overall impression [44]. Members of the research team have extensive training and experience designing, developing, and testing innovative eHealth technologies [28,31,38,41,45].

## Methods

### Aim 1

#### Study Design, Setting, and Context

A user-centered design is presently being conducted at SHC-Canada, a bilingual (French and English), university-affiliated orthopedic hospital, inviting input on a global scale. Adoption of a user-centric design and a “user experience of websites” conceptual framework [46,47] will guide the collaborative and participatory design and development of Teens OI, addressing prior limitations including developing eHealth interventions without explicit theoretical frameworks, using single measures with limited end user input, and neglecting environmental factors [48]. By partnering with the end users (ie, youths with all OI Types and their parents) from study onset [49], we will lay the foundations for an effective eHealth program and ensure the content and format of the program are relevant, acceptable, and culturally sensitive [16,35,39,40,50]. Aligned with Shriners’ Mission and FOCUSED philosophy of care [51,52], we are seeking to close the transitional care gap encountered by youths with OI and their families, improve the quality of health care services, and help youths and their families better manage OI. Our analysis of all education material for patients with OI at SHC-Canada revealed the dearth of resources available, targeting solely the parents of patients with OI [53]. Hence, our team has partnered with the end users from the study onset with the goal of introducing a series of eHealth programs to complement our existing health care services and enhance the global reach of Shriners [12,54].

#### Participants

A “Website Design and Development Council” (ie, Council) has been convened. Membership comprises patients, parents, health professionals, and decision makers (n=8-12) from the study site and the greater OI community. Maximum variation sampling techniques were used to recruit 20 youth-parent dyads from SHC-Canada over 5-10 cycles. The inclusion criteria consisted of (1) youths between 12 and 21 years of age; (2) youths receiving OI care at the study site; and (3) both youths and parents able to speak and read English or French. The exclusion criteria excluded youths if they had major cognitive impairments or comorbid medical or psychiatric illnesses that may impact their ability to understand and use the program. The power of the maximum variation sampling technique used lies in selecting information-rich cases for in-depth study, which maximizes variation and diversity. The variability affords a more comprehensive understanding of the many diverse experiences that will enhance the design and development of Teens OI and the transferability of the proposed eHealth platform. While a sample size of 20 youths and parent dyads was proposed, the sample size was tentative and may change based on achieving data saturation and the desire for a diverse sample (eg, cultural, linguistic, OI type, and severity of disability) [55,56]. About 150 eligible patients with OI were followed at the study site. Our experiences suggest there is substantial interest in study participation, with over >90% of participation rated as experienced at the study site [22]. Our youths and their parents remain concerned about their impending transition and welcome the proposed support [12,14,16].

#### Recruitment

A multipronged approach was used to announce the study and commence recruitment (Multimedia Appendix 1). To protect the families’ privacy, the health care team assisted by identifying, screening, and approaching the families to determine if they were interested in hearing more about the study. If the patients express interest in hearing more about the study, their names and contact information are provided to the clinical research coordinator, who approaches the patients, provides a verbal and written explanation of the study, and, if agreeable, obtains written informed consent for study participation and audio-taping. Parents consent for their child to participate, and they consent for themselves to participate. Youths aged 12-13 years, provide assent and youths aged 14-21 years provide consent (Multimedia Appendix 2). Each study participant receives a copy of the consent. The health care professionals consider patients’ health status and interest in participating in the study during regularly scheduled clinic appointments and in-patient visits. The recruitment method will be supplemented, if needed, by sending a study postal information letter, signed by their medical doctor and principal investigator, inviting them to participate at home.

#### Procedures

The Council will oversee the design and development of Teens OI using Garrett’s Theory of User Experience [47]. Garrett proposed the following 5 different planes of a website that affect the user experience: surface, skeleton, structure, scope, and strategy, which have been used for understanding and improving youths’ exposure to internet-delivered interventions [46,47]. The Council will receive training in patient engagement and partnerships, which includes topics of cultural competency [57], mutual respect, and inclusivity [58]. Conceptboard, a centralized hub for content and ideas for visual projects, will be used to facilitate the web-based, real-time, collaborative design and development of Teens OI with the global OI community and eliminate social distancing barriers due to COVID-19 [44]. Varying data will be collected in English and French by conducting youths’ and their parents’ interviews and focus groups; hosting 2 symposiums at SHC-Canada; presenting ongoing work; soliciting input from OI experts; and surveying the OI community using Qualtrics. Presently, we foresee 12 interactive, multicomponent modules adapted from Teens JIA [28] being designed for our diverse English- and French-speaking populations. Although draft modules have been created, we will inquire about topics and create, modify, or eliminate draft modules as needed. Other features such as multimedia (eg, video and audio) and other interactive, technological features (eg, discussion boards, animations, quizzes, stories of hope, and video clips) will be designed and developed with input from the end users.

#### Instrumentation, Interview, Focus Group, and Symposia Guides

Guided by Garrett’s 5 planes contributing to users’ experiences of websites [47], interview guides (Multimedia Appendix 3) outlining the procedures, questions, and probes have been created to guide discussions of website development and design, user experiences, and tools to solve related problems (1-2 cycles per plane). While each plane will be addressed individually, the discussion will naturally combine all planes. Field notes and feedback from the first cycle of interviews and focus groups will inform the discussion for subsequent cycles of data collection. With 117 user experience instruments available, the Council will decide on the quantitative metrics of the design and development of Teens OI [59]. One sociodemographic survey will be used to collect demographic, health, and internet-use characteristics [12,16].

#### Data Management, Analysis, and Interpretation

All survey data are being descriptively analyzed using SAS (version 9.4; SAS Institute Cary). Observations, field notes, and transcribed data will be imported into NVivo (version 12; QSR International Pty Ltd) for data management and analysis. The qualitative data are analyzed using content analysis techniques involving an iterative process of data reduction, data display, conclusion drawing, and verification [60]. All data will be analyzed and triangulated in each of the 5 planes, fostering the process of “building from the bottom to the top” and “moving from abstract to concrete,” allowing for refinement of Teens OI between each cycle within each of the 5 planes [47]. Individual and aggregate comparisons and contrasts of subgroups (eg, parent or youth [22,61,62]; English or French; OI Types and disability severity) are lending insight into how to synergize results and establish consensus with participants and the Council to maximize the inclusivity of Teens OI. An audit trail, composed primarily of methodological and analytical documentation, will be kept to permit the transferability of this innovative process [63]. Unanimous support from the Council to proceed to Aim 2 will be sought.

### Aim 2

#### Study Design, Setting, and Context

A user-centered, mixed methods, concurrent design will be used to test usability. Usability testing is critical to ensure content relevancy; determine if the eHealth program is easy to use and efficient to complete; and confirm the end user product acceptability [28,31,38,41,45]. Using varying data sources, the usability testing will ensure the end users are able to access, understand, and use the Teens OI content and that the Teens OI can be delivered in an efficient, effective, and satisfying manner. The study setting and context will be the same as in aim 1.

#### Participants

Maximum variation sampling techniques will be used to recruit up to 32 youth-parent dyads from SHC-Canada. The inclusion and exclusion criteria will be the same as described in aim 1. Usability testing produces informative results with small sample sizes [64], with 80% of usability problems detected with 4 or 5 participants and 95% of problems identified with 9 participants [65,66]. Previous usability studies of Teens Taking Charge included sample sizes of 18 [31], 19 [28], 21 [41], and 37 [38]. Iterative rounds of testing with 8 youths per round will be conducted until 95% of usability issues have been determined.

#### Recruitment

The process for recruitment will be the same as that in aim 1.

#### Procedures

The usability testing will occur in 4 iterative cycles to assess the user performance and level of satisfaction of Teens OI and determine the need for refinement after each cycle. Consenting participants (8 in each of the 4 cycles) will complete a sociodemographic questionnaire, followed by a one-on-one observation recorded with Morae 3.3 (TechSmith Corp) usability software, and a semistructured interview with the research assistant (RA). Participants will access the website via a laptop computer for use at the point of care. Morae, activated at the beginning of instruction, will be used for the automatic recording of user performance and level of satisfaction. Once the automatic recordings have begun, the RA will direct the participants to the main navigation menu and prompt them to select one of the modules of interest to them. The RA will use a standardized script to instruct participants to select one or more key features or functions within the selected module. Participants will be encouraged to “think aloud” and comment on any difficulties, as they explore various features and functions within each module and complete various usability tasks (eg, select a module from the main navigation menu, click on a video, and complete an exercise). Scripted instructions will continue until the participant has viewed all of the modules or shows disinterest in exploring further. After each cycle iteration, changes to the website will be made accordingly, and usability testing will continue until no further significant issues have been identified. Participants will be instructed that they can stop participating in the study at any time. They will also have opportunities to debrief, ask questions, offer feedback, and receive assistance as needed. During the testing, the RA will also record their observations and take field notes. It is anticipated that the interviews will take 45-60 minutes, with length varying depending on the participants**’** schedules and interests.

#### Measures and Standardized Scripts

Guided by the Health IT Usability Evaluation Model (Health-ITUEM) [67,68], standardized scripts outlining the procedures, questions, and probes will be adapted from previous Teens Taking Charge research and used [28,31,38,41]. Usability surveys will also be used [69,70]. The sociodemographic survey in aim 1 will be used [12,16].

#### Data Management and Analysis

All sociodemographic data will be imported into SAS 9.4 for descriptive statistical analysis. Recorded Morae data will be downloaded, coded, and analyzed using descriptive statistics. Observations, field notes, and transcribed data will be imported into NVivo 12 for data management and analysis. The qualitative data will be analyzed using content analysis techniques involving an iterative process of data reduction, data display, conclusion drawing, and verification [60]. All data will be analyzed in 2-4 cycles to allow for prototype refinement between each cycle. Teens OI will also be compared to other OI websites using relevant evaluation indexes from the Alexa website ranking system [71] and Google Analytics [72]. An audit trail, composed primarily of methodological and analytical documentation, will be kept to permit the transferability of this innovative process [63]. As a benchmark of success, the team will strive to resolve 95% of problems, and have mean System Usability Scale and Health Information Technology Usability Evaluation Scale scores of 70% corroborated with positive findings.

### Ethics Approval

This minimal-risk study has received feasibility, scientific, and administrative approvals from the Department of Research Programs at SHC International Headquarters and ethical approval from the institutional research ethics board (phase 1: A04-B31-21B, and phase 2: A10-B97-22B). Most participants for study inclusion (n=150) have been followed at the study site for several years, with follow-up visits at least biannually. The burden is relatively small, with no additional visits required for participation. While study participation is not associated with a greater risk than expected or encountered in daily life, distress from discussing self-management needs or preparing for transitioning into the adult health care system may occur. If periods of increased distress arise for the participants, data collection sessions will be postponed and resumed at a later time. Similarly, at any time during or after the study, the participants are free to decline to answer any question that makes them uncomfortable, to stop the interview, or to withdraw from the study. Instructions on how to withdraw are also provided to the participants and their parents during the informed consent discussion. If our team is experiencing any problems during the course of study, we will revisit as a team to strategize, offer solutions, or allocate resources as deemed necessary. Presently, the alternative to participating in the study is to not participate and to seek information and support for self-management and transition from published materials available for free or for purchase (eg, books), attend caregiver programs that exist in the community, or seek help from physicians, mental health practitioners, or other allied health care providers that exist in the community. If a potential participant screens out or does not want to participate but requests information about resources, we will refer the individual to the OI clinical team with their permission.

### Knowledge Dissemination

The research team is actively presenting the research in progress at varying meetings to share and solicit input from the global OI community. Preliminary findings have been presented at 2 international meetings [73,74] and awarded a New Investigator Award. Feedback received is being shared and discussed with the Advisory Council, which may influence the research process, including data analysis, interpretation, and discussion of findings, or the content creation process of Teens OI. The findings will be subsequently published in peer-reviewed journals and mobilized via the global community network through social media, newsletters, and community, and academic events.

## Results

Aim 1 received ethical approval on June 28, 2021. As of December 2021, an 8-person, interdisciplinary Teens OI council, comprised of 4 health care professionals (nurse practitioner, care coordinator, nurse, and social worker), 3 youths and young adults with OI, and 1 parent, has been convened to oversee the design and development of Teens OI. Two cycles of interviews have been conducted with 10 youths with OI with or without their parents (n=6) from December 2021 to September 2022. There have been no withdrawals. Attrition rates, including dropouts and those lost to follow-up, may range from 10% to 15% (which is similar to Teens JIA [32] and considered low compared to other internet-based studies in adolescents (0-28, mean 14.3) and adults, in which higher dropout rates were found [9,32]). Data analysis has been in progress since April 2022. Aim 2 is ethically approved and will commence following the completion of content development, expected by late July 2023. Preliminary analysis indicates that the following topics need to be prioritized for the youths: mental health, pain, accessibility, medical care, education, community, and parental care.

## Discussion

### Principal Findings

The survival of youths with chronic health conditions into adulthood has created a critical demand for effective self-management and transitional care programs to optimize the quality of care and quality of life of children with rare diseases worldwide. SHC-Canada, internationally acclaimed for establishing the worldwide standard of care for children with OI, is of no exception. Ongoing, multistakeholder evaluation of transitional care services at SHC-Canada has identified the need for a web-based self-management program to better prepare patients with OI and their families for transition into the adult health care system. Preliminary work consisting of needs assessments, knowledge syntheses, and symptom assessments has ensued to inform the creation of such an evidence-based program with priority topics identified. A long-standing partnership with Stinson, who has led similar studies (registered on clinicaltrials.gov) with youths with arthritis (ID NCT01572896), cancer (ID NCT02299219), hemophilia (ID NCT01477437), and organ transplants, has subsequently catalyzed our efforts to offer Teens OI. Collectively, our interdisciplinary research team is uniquely positioned to address the transitional care gap in the OI community. Our team has led studies of self-management and transitional care in OI [14,16-18] and begun developing eHealth innovations (ie, OI Good to Go Passport [17] and Sisom OI [47]). However, the need for this Teens OI is global. After presenting our in-progress work at 2 international OI meetings [73,74], there is a global request to merge our efforts, knowledge, and expertise. To further catalyze efforts and mobilize the community to enhance the paucity of resources available for this rare disease patient population, we encourage interested individuals, patient organizations, and institutions from the public, private, and industry sectors to express their interest and reach out to the team. A significant amount of content needs to be created amidst the dearth of evidence available in OI, especially tailored to the youth audience. We welcome the sharing of resources, especially any gray source material (eg, pamphlets, policies, tools, pictures, graphics, videos, or teaching materials), to determine how to ideally integrate into Teens OI and to recognize the contributions of others. Moreover, translators will be needed to render Teens OI accessible to communities unable to comprehend English or French and to collaborate on tailoring Teens OI to the respective languages, cultures, and contexts.

### Troubleshooting

Due to COVID-19 restrictions imposed at the study site related to large crowd gatherings, our research team opted to solidify global input on our in-progress findings at 2 international OI meetings to date [73,74]. This warranted an ethical amendment allowing us to survey the global OI community with questions generated from our in-progress design analysis [73]. Once large-scale, in-person meetings are reconvened, the research team will host a symposium related to self-management and transitional care at the study site to further inform the content creation process. Software restrictions in the study have precluded the use of Conceptboard. Hence, this innovative feature permitting real-time, collaborative design and development of Teens OI with the global OI community was not used. Rather, design features are transferred to PowerPoint slides shared via Microsoft Teams in individual or focus groups for feedback.

### Strengths and Limitations

A rigorous and innovative approach is being used to design, develop, and test the usability of Teens OI*.* This self-management and transitional care program will be designed, developed, and tested in partnership with youths with OI, their parents, and the greater OI community. This user-centric approach will advance the neglected OI field of research, align with the mission and philosophy of care (FOCUSED) of the study site, and capitalize on youths as early adopters of eHealth technologies to meet their needs. Findings will generate the provision of the Teens OI eHealth prototype, which will be ready for feasibility testing and outcome evaluation. The development of Teens OI will address a critical gap in the quality of care and our growing concerns that the OI population transitioning from our study site and other pediatric hospitals is at risk of various adverse events in terms of morbidity, mortality, social, educational, and vocational outcomes. Moreover, this research program will address a serious lack of health care resources designed with patients to improve the quality of care and life. Finally, actively engaging our patients in the provision of their care is their fundamental right, and it is our duty as health care professionals to offer these quality services. Presently, the findings generated from this study will be primarily derived from English- and French-speaking youths, parents, and clinicians. Efforts are in place to seek evidence (eg, documents, videos, and input) from the global community to guide the design and development of content. While the participants are from varying ethno-cultural backgrounds, they are primarily from Canada and treated at a study site internationally renowned for the treatment of OI. The transferability of the Teens OI prototype will need to be assessed and tailored to other contexts accordingly.

## Appendix

Multimedia Appendix 1 Recruitment Flyers.

Multimedia Appendix 2 Assent and Consent Forms.

Multimedia Appendix 3 Interview Guides.

Multimedia Appendix 4 Peer review report by Shriners Hospitals for Children.

## Abbreviations

JIA: juvenile idiopathic arthritis
OI: osteogenesis imperfecta
RA: research assistant
SHC-Canada: Shriners Hospitals for Children-Canada
Teens OI: Teens Taking Charge: Managing OI Online

## Data Availability

Congruent with the study site policies, any unique research resources developed through SHC-sponsored research will be available to individuals upon request from any research team member. These resources may include consent forms, instruments, interview guides, databases, coding frameworks, methods, the Teens OI prototype, and dissemination material. These resources will be available for research purposes to qualified individuals within the scientific and OI communities through academic and nonacademic publications and consultations.
