# Supplementary material for: The Design, Development, and Usability Testing of an eHealth Program for Youths With Osteogenesis Imperfecta: Protocol for a 2-Phase User-Centered Mixed Methods Study
Source: JMIR Res Protoc. 2023 Jun 23;12:e47524. doi: 10.2196/47524 (PMC10337436; doi:10.2196/47524)
Supplement: Multimedia Appendix 1 [file resprot_v12i1e47524_app1.pdf]

Teens OI Phase I: Telephone Recruitment Script

CRC: Hello, may I speak with

- *If 14y+:* [name of potential participant]
- *If aged less than 14y:* the parents or legal guardians of [name of potential participant]

My name is *[insert name]* and I am a research coordinator at the Shriners Hospitals for Children in Montreal. I am calling to ask you whether you would be interested in participating in a research study that is being carried out right now for youth with Osteogenesis Imperfecta and their parents. Are you interested in hearing more about the study?

****if they say NO, thank them for their time and hang up. If they say yes, continue:***

The purpose of this study is to design and develop an e-health program to help youth self-manage their OI and prepare for their future transition to the adult-health care system. As a study participant, you would help us develop the Teens OI website by participating in interviews or group meetings. Does this sound like something that may interest you?

****if they say NO, thank them for their time and hang up. If they say yes, continue:***

Great! I will go over the consent form with you either in person, at your next scheduled hospital visit on [insert date] or over the phone at a time that is convenient for you. Which option do you prefer?

If they prefer in person, agree on a time and date. Ask for their email to send them a copy of the consent forms in the meantime.

Date/Time:______________ **Email:**_______________________________________

If they prefer to provide verbal consent over the phone, agree on a time and date for the ICF discussion. Ask for their email to send them a copy of the consent forms in the meantime.

Date/Time:______________ **Email:**_______________________________________

follow the ICF template if verbal consent will be provided.

Thank you for your interest in participating in the study. We are excited to develop and design the Teens OI website with you.

Need Expert Advice from Parents of Youth with OI

Helping our Youth Self-Manage and Transition to the Adult-Oriented Health Care Systems

Dropped in the Jungle
with No One to Call

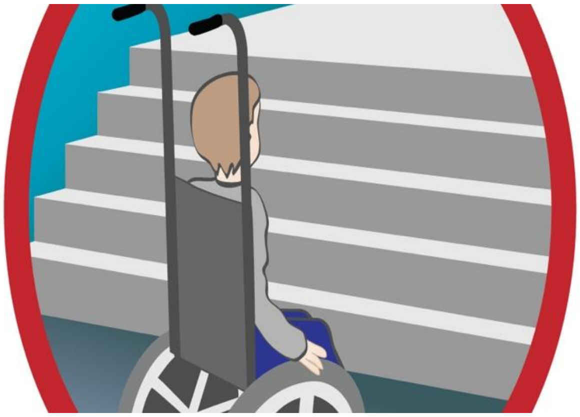

They Don't Know
How to Treat Me!

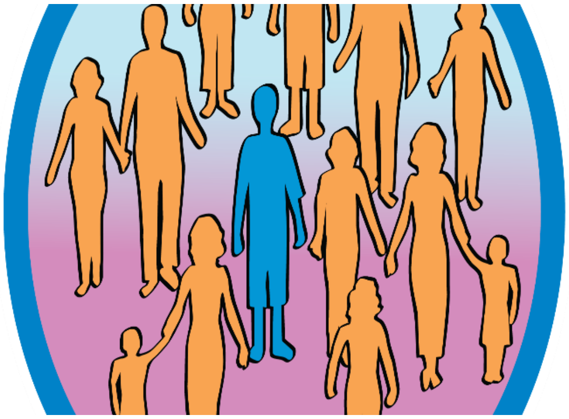

"And you know, I've met how many doctors that don't even know what OI is." [Father of a Youth with OI]

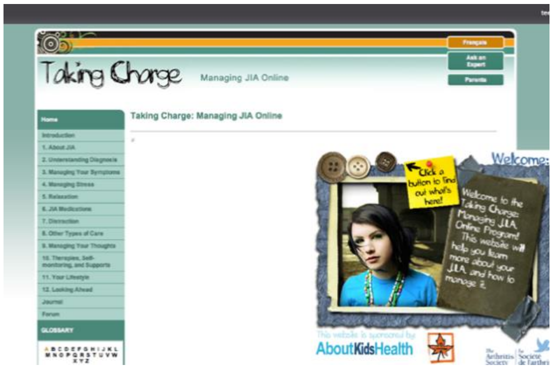

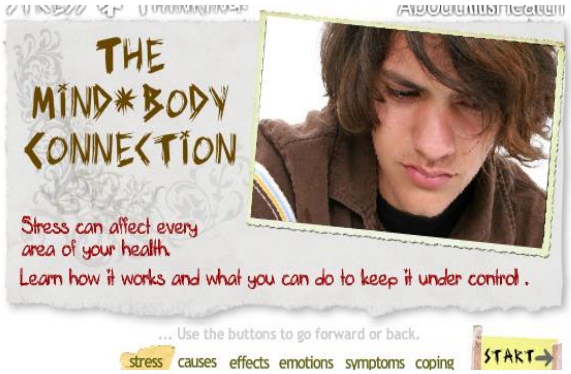

For more information, please contact:

Sofia Addab, MSc
Clinical Research Coordinator
Shriners Hospitals for Children ® – Canada
Telephone: 514-842-4464 ext. 2264
E-mail: sofia.addab@shrinenet.org

Argerie Tsimicalis, RN, PhD
Principal Investigator
Nurse Scientist, Shriners Hospitals for Children ® – Canada
Associate Professor, McGill University
Telephone: 514-842-4464 ext. 2268
(Cellular): 514-770-6069
E-mail: atsimicalis@shrinenet.org
