# Supplementary material for: The Design, Development, and Usability Testing of an eHealth Program for Youths With Osteogenesis Imperfecta: Protocol for a 2-Phase User-Centered Mixed Methods Study
Source: JMIR Res Protoc. 2023 Jun 23;12:e47524. doi: 10.2196/47524 (PMC10337436; doi:10.2196/47524)
Supplement: Multimedia Appendix 2 [file resprot_v12i1e47524_app2.pdf]

Hôpitaux Shriners pour enfants®
Shriners Hospitals for Children®
Canada

Shriners Hospitals for Children®-Canada

INFORMED CONSENT AND AUTHORIZATION
TO TAKE PART IN A RESEARCH PROJECT OR STUDY (COUNCIL: HCPs + DM)

**TITLE:**	Usability Testing of an E-Health Program for Youth with Osteogenesis Imperfecta (Phase II)
**SHRINERS PROTOCOL NO.:**	CAN2105
**IRB APPROVAL NO.:**	*TBD*
**PRINCIPAL INVESTIGATOR:**	Argerie Tsimicalis, RN PhD<br>1003 Décarie Blvd. Suite 2.19<br>Montreal, Quebec, H4A IM0<br>Canada<br>(514) 842-4464 ext. 2268<br>atsimicalis@shrinenet.org
**CLINICAL RESEARCH COORDINATOR:**	Sofia Addab, MSc<br>1003 Décarie Blvd. Suite 2.19<br>Montreal, Quebec, H4A IM0<br>Canada<br>(514) 842-4464 ext. 2264<br>sofia.addab@shrinenet.org

PARTICIPANT: ____

SUMMARY
Research studies include only people who want to take part. The purpose of this informed consent form is to help you decide if you want to be in the research study.

Before you decide if you want to take part, it is important that you read and understand this consent form. Please take your time to make your decision. Please ask questions about anything that you do not understand before deciding whether to participate.

The person in charge of this study is Argerie Tsimicalis, RN Ph.D. There may be other people on the research team helping during the study.

Why are you being asked to take part in this study?

You are being asked to take part in this research study because you deliver care to patients with OI or are a decision-maker involved in the care of patients with OI at the Shriners Hospitals for Children®-Canada.

The purpose of the study is to test the usability of a program called "*Teens Taking Charge: Managing OI Online" (Teens OI).* This is an interactive, e-health program designed to help youth learn about their OI, manage their condition, and offer social support. There is also a section for parents.

The information you provide us will help develop an effective, user-friendly, Internet-based, self-management program for youth with OI to help them better cope with OI, improve their health-related quality of life, and enhance their readiness to transition to adult health care. It will also help parents prepare for their child's transition.

How many people will take part in the study?

The Council will consist of 8-12 individuals, which may include patients, young adults with OI, parents, healthcare professionals, or decision-makers.

An additional 32 youths with OI and 32 parents will participate in the study and help as well in the usability testing of the website.

What will happen in this study?

If you can be in the study, and you choose to take part, you will be part of a Council that will oversee the usability testing of the Teens OI website. You will participate in four focus groups (1-hour each, for a total of 4 hours) with other members of the Council. The focus groups may be done in-person or online using the Microsoft Teams platform. In the event the focus groups will be online, we will schedule a time and date, and send you the link via the email you provide to us. With your permission, we would like to audio-record the focus group discussions.

In the first focus group, you will receive training in patient engagement and partnerships, brainstorm usability questions, and identify OI experts that should be invited to the final symposium.

During the second and third focus groups, you will review feedback received from youth with OI and their parents on the usability of the website and make suggestions to make the website more user-friendly. You will provide feedback on the preparation for the Symposium and be invited to attend the event.

During the last focus group, you will be invited to finalize the website based on the findings from the Symposium and approve any final changes.

What will happen in this study that is "research"?

There are no experimental tests used in this study.

How long does the study last?

Each focus group will last about 1 hour, for a total of 4 hours. The final Symposium will be developed with input from the Council. This event may be for a few hours to a whole day. You may decide at a later point if you would like to attend the Symposium. Overall, the study will take place over a 9-12 month period.

Can you stop being in the study?

Yes. You can drop out of the study at any time. No one will be upset. It will not affect your employment at the hospital. You can do this by telling one of the members of the research team.

There are no anticipated circumstances under which we would decide to take you off the study.

What are the risks of the study?

This study involves no more than minimal risks, which means that we expect that you will have no more risk than those you have in your normal daily life.

Are there benefits to taking part in the study?

You will not directly benefit from participating in this study. Your feedback will be used to inform the usability of the online self-management program for adolescents with OI, which may help adolescents to better manage their OI. If you would like to provide your contact information (email or mailing address) we will send you information on the study findings and updates on the online self-management program.

What other options are available to you?

If you decide not to enter this study, your alternative is not to be in this study.

How will information about you be kept private?

You will not be identified by name or other personal identification. To protect your identity, the research team will only use code numbers to refer to your information. A list linking the code number with your name will be stored in a separate file. All data, with identifying information removed, will be entered and securely stored into a data management system.

If information from this study is published or presented at scientific meetings, your name and other personal information will not be used

Data will be kept for seven years following the publication of the study results. Subsequently, paper documents will be shredded, and electronic data will be destroyed by secure deletion according to the Retention and Destruction policy at Shriners Hospital for Children.

What are the costs?

You will not be paid for being in this study. There are no costs to take part in this study.

What are your rights if you take part in this study?

Voluntary Participation: Taking part in this study is your choice. You may choose not to take part in the study or you may leave at any time. Your decision about being in this study will not affect your employment at Shriners Hospitals for Children®-Canada. If you have any questions about the study, you can always talk to one of the study staff. Do not sign this form unless you have had the chance to ask questions and have received clear answers.

New Findings: We do not expect to find any new information that may affect your willingness to stay in this study during your brief involvement. However, we will tell you if there were any new findings.

Whom can you call if you have questions or problems?

If you have any questions, please ask us. If you have any questions later, please contact Dr. Argerie Tsimicalis by telephone at (514) 842-4464 ext. 2268 or by email at: atsimicalis@shrinenet.org. You can contact the Ethics Officer for the McGill IRB, Ms. Ilde Lepore, by telephone at (514) 398-8302 or by email at ilde.lepore@mcgill.ca for answers to questions you might have about research and your rights as a research participant.

Title of Research Project: Usability Testing of an E-Health Program for Youth with Osteogenesis Imperfecta (Phase II)

Your signature below will show that you acknowledge and agree that:

- **You have read this consent form (or it has been read to me)**
- **You will freely take part in this research study,**
- **You have had a chance to ask questions and all of your questions have been answered**
- **You have read and understood the information above**
- **By signing this consent form, you have not given up any of my legal rights.**

You will be given a signed and dated copy of this informed consent form which is yours to keep.

CONSENT SIGNATURE:

____ ____________
Signature of Participant Date

Email: ____

....

Using language that is understandable and appropriate, I have discussed this project and the items listed above with the participant and/or his parent/legal guardian.

____ ______________________
Signature of the person who conducted the informed consent discussion Date

____ ______________________
Print Name Study Role

Hôpitaux Shriners pour enfants®
Shriners Hospitals for Children®
Canada

Shriners Hospitals for Children®-Canada

INFORMED CONSENT AND AUTHORIZATION TO TAKE PART IN A RESEARCH PROJECT OR STUDY (COUNCIL: youth, YA, parents)

**TITLE:**	Usability Testing of an E-Health Program for Youth with Osteogenesis Imperfecta (Phase II)
**SHRINERS PROTOCOL NO.:**	CAN2105
**IRB APPROVAL NO.:**	*TBD*
**PRINCIPAL INVESTIGATOR:**	Argerie Tsimicalis, RN PhD<br>1003 Décarie Blvd. Suite 2.19<br>Montreal, Quebec, H4A IM0<br>Canada<br>(514) 842-4464 ext. 2268<br>atsimicalis@shrinenet.org
**CLINICAL RESEARCH COORDINATOR:**	Sofia Addab, MSc<br>1003 Décarie Blvd. Suite 2.19<br>Montreal, Quebec, H4A IM0<br>Canada<br>(514) 842-4464 ext. 2264<br>sofia.addab@shrinenet.org

PARTICIPANT: ____

When we say "you" in this informed consent form, we mean you or your child; "we" means the doctors and research staff.

SUMMARY

You are being asked to take part in a research study. The purpose of this informed consent form is to help you decide if you want to be in the research study.

Research studies include only people who want to take part. Before you decide if you want to take part, it is important that you read and understand this consent form. Please take your time to make your decision. Discuss it with your parents, friends, and family. We encourage you to include your parents in the discussion and decision. Please ask questions about anything that you do not understand before deciding whether or not to participate.

The person in charge of this study is Dr. Argerie Tsimicalis. There may be other people on the research team helping during the study.

Things to know and understand before deciding to take part in a research study:

- The main goal of a research study is to learn things to help patients in the future.
- The decision to join or not join the research study will not cause you to lose any medical benefits. If you decide not to take part in this study, your doctor will continue to treat you.
- After reading the consent form and having a discussion with the research staff, you should know which parts of the study are experimental and which are standard medical care.

Why are you being asked to take part in this study?

You are being asked to take part in a study because you are a teen who has been diagnosed with OI and will be, in the future, transitioning to the adult health care system, or are a young adult with OI, or are the parent of a teen with OI.

The purpose of this study is to gather your input on the usability of a program called "*Teens Taking Charge: Managing OI Online" (Teens OI).* This is an interactive, e-health program designed to help youth learn about OI, manage their condition, and offer social support. There is also a section for parents.

The information you provide us will help develop an effective, user-friendly Internet-based self-management program for youth with OI to help them better cope with OI, improve their health-related quality of life, and enhance their readiness to transition to adult health care. It will also help parents prepare for their child's transition.

How many people will take part in the study?

The Council will consist of 8-12 individuals, which may include patients, young adults with OI, parents, healthcare professionals, or decision-makers.

An additional 32 youth with OI and 32 parents will participate in the study and help as well in the usability testing of the website.

What will happen in this study?

If you can be in the study, and you choose to take part, you will be part of a Council that will oversee the usability testing of the Teens OI website. You will participate in four focus groups (1-hour each, for a total of 4 hours) with other members of the Council. The focus groups may be done in-person or online using the Microsoft Teams platform. In the event the focus groups will be online, we will schedule a time and date, and send you the link via the email you provide to us. With your permission, we would like to audio-record the focus group discussions.

In the first focus group, you will receive training in patient engagement and partnerships, brainstorm usability questions, and identify OI experts that should be invited to the final symposium.

During the second and third focus groups, you will review feedback received from youth with OI and their parents on the usability of the website and make suggestions to make the website

more user-friendly. You will provide feedback on the preparation for the Symposium, and be invited to attend the event.

During the last focus group, you will be invited to finalize the website based on the findings from the Symposium, and approve any final changes.

What will happen in this study that is "research"?

There are no experimental tests used in this study. Your prospective participation in the Council focus groups is part of the research study.

How long does the study last?

Each focus group will last about 1 hour, for a total of 4 hours. The final Symposium will be developed with input from the Council. This event may be for a few hours to a whole day. You may decide at a later point if you would like to attend the Symposium. Overall, the study will take place over a 9-12 month period.

Can you stop being in the study?

Yes. You can drop out of the study at any time and no one will be upset. It will not affect your other care and treatment received at Shriners Hospitals for Children. You do this by contacting the study coordinator, Sofia Addab, at 514-842-4664 ext 2264 or by email at sofia.addab@shrinenet.org. It is important to know that there will be no negative impact if you choose to stop.

If you drop out of the study, no new health information identifying you will be gathered after that date. Information that has already been gathered may still be used and given to others. Additionally, if you choose to participate but do not complete study procedures, you may be dropped from the study.

The researcher conducting this study may decide to take you off this study without your consent under the following circumstances:

- If she or he believes that it is in your best interest
- If study procedures are not followed or if the focus group attendance is not met

What are the risks of the study?

This study involves no more than minimal risk, which means that we expect that you will have no more risk than those you have in your normal daily life or routine physical (*or psychological*) examination or tests.

Are there benefits to taking part in the study?

You will not directly benefit from participating in this study. Your feedback will be used to inform the usability of the online self-management program for adolescents with OI, which may help other adolescents and parents to better manage OI. If you would like to provide your contact

information (email or mailing address), we will send you information on the study findings and updates on the online self-management program.

What other options are available to you?

This is not a treatment study. Your alternative is not to be in this study.

What are the costs of taking part in this study?

You will not be paid for being in this study.

In the case of injury or illness resulting from this study, medical treatment is available. Please notify the investigator as soon as possible if you need to seek medical care for an injury or illness resulting from this study. Although Shriners Hospitals for Children is not able to offer financial compensation for an injury or illness resulting from this study, to the extent Shriners Hospitals for Children provides the needed medical treatment at its facility, that treatment may be provided at no cost to you. If you obtain that care somewhere else, your usual healthcare coverage may apply. Although Shriners Hospitals for Children has not set aside any funds to pay you for injury or illness, you do not give up any of your legal rights by signing this form.

What are your rights if you take part in this study?

Voluntary Participation: Taking part in this study is your choice. You may choose not to take part in the study, or you may leave at any time. Your decision about being in this study or refusal will not affect your care at Shriners Hospitals for Children. If you have any questions about the study, you can always talk to one of the study staff. Do not agree to take part in the study unless you have had the chance to ask questions and have received clear answers.

New Findings: We do not expect to find any new information that may affect your willingness to stay in this study during your brief involvement. However, we will tell you if there were any new findings.

Who will be paying for the study?

Shriners Hospitals for Children, the sponsor, will pay for this study.

Whom can you call if you have questions or problems?

If you have any questions, please ask us. If you have any questions later or in the event of research-related injury, please contact Dr. Argerie Tsimicalis by telephone at 514-842-4464 ext. 2268 or by email at: atsimicalis@shrinenet.org. You can contact the Ethics Officer for the McGill IRB, Ms. Ilde Lepore, by telephone at (514) 398-8302 or by email at ilde.lepore@mcgill.ca for answers to questions you might have about research and your rights as a research participant.

It is important that you tell your study doctor if you feel that you have been injured because of taking part in this study. In the event of research-related injury, please call Ms. Sofia Addab at (514) 842-4464 ext. 2264

AUTHORIZATION TO USE AND DISCLOSE INFORMATION FOR RESEARCH PURPOSES

What information may be used and given to others?

No personal health information (PHI) will be used or disclosed.

Who may use and give out information about you in connection with this research study?

- The principal investigator, Dr. Argerie Tsimicalis;
- Other members of the research team affiliated with Shriners Hospitals for Children who might need to use or disclose information from your study file in connection with this study.

Who might get this information?

The people mentioned above may share or disclose information from your study file with the following entities or people in connection with a research study.

- Shriners Hospitals for Children, the sponsor of this research. “Sponsor” means any persons or companies that are working for or with Shriners Hospitals for Children
- The McGill Faculty of Medicine Institutional Review Board
- Regulatory authorities such as Health Canada

How will information about you be kept private?

You will not be identified by name or other personal identification. To protect your identity, the research team will only use code numbers to refer to your information. A list linking the code number with your name will be stored in a separate file. All data, with identifying information removed, will be entered and securely stored into a data management system.

We will do our best to make sure that the personal information in your study file will be kept private. However, absolute confidentiality cannot be guaranteed because of the need to give information to the above-mentioned parties. Your personal information may also be given out if required by law, such as pursuant to a court order.

Data will be kept for seven years following the publication of the study results. Subsequently, paper documents will be shredded and electronic data will be destroyed by secure delete according to the Retention and Destruction policy at Shriners Hospital for Children.

Why will this information be used and/or given to others?

- To do the research in this study,
- To examine results related to this study, and
- To see if this study was done right

The results of this research study may be presented at meetings or in publications. Your name and other personal information will not be used in those presentations.

What if you decide not to give permission to use and give out your health information?

Then you may still be able to be in this research study, but the results may be less useful for this research.

May you review or copy your information?

Yes, but only after the research is over.

May you withdraw or revoke (cancel) your permission to use and give out your health information?

This permission will be good until December 31, 2070.

You may withdraw or take away your permission to use and disclose your health information at any time. You do this by sending a written notice to the study doctor. If you withdraw your permission, you will not be able to stay in this study.

When you withdraw your permission, no new health information identifying you will be gathered after that date. Information that has already been gathered may still be used and given to others.

Is your health information protected after it has been given to others?

There is a risk that your information may be given to others without your permission. Any information that is shared may no longer be protected by federal privacy rules.

Title of Research Project: Usability Testing of an E-Health Program for Youth with Osteogenesis Imperfecta (Phase II)

Your signature below will show that you acknowledge and agree with the following statements:

- **I have read this consent form (or it has been read to me)**
- **I will freely take part in this research study,**
- **I have had a chance to ask questions and all of my questions have been answered**
- **I have read and understood the information above**
- **By signing this consent form, I have not given up any of my legal rights.**

You will be given a signed and dated copy of this informed consent form which is yours to keep.

CONSENT SIGNATURE:

____ __________
Signature of Participant Date

OR

_________________________________ __________ ________________________
Signature of Parent or Subject's Legally Authorized Representative Date Relationship to Participant

Email: ____

....

Using language that is understandable and appropriate, I have discussed this project and the items listed above with the participant and/or his parent/legal guardian.

____ _____________________
Signature of the person who conducted the informed consent discussion Date

____ _____________________
Print Name Study Role

ASSENT SECTION:

Statement of the person conducting assent discussion:

- I have explained all aspects of the research to the subject to the best of his or her ability to understand.
- I have answered all the questions of the subject relating to this research.
- The subject agrees to be in the research.
- I believe the subject's decision to enroll is voluntary.
- The study doctor and study staff agree to respect the subject's physical or emotional dissent at any time during this research when that dissent pertains to anything being done solely for the purpose of this research.

Statement of Parent or Guardian:

My child appears to understand the research to the best of his or her ability and has agreed to participate.

____ ____________________
Signature of Parent or Guardian Date

❑ I have explained the study to the extent compatible with the subject's capability, and the subject has agreed to be in the study.

OR

❑ The subject is not able to assent because the capability of the subject is so limited that the subject cannot reasonably be consulted.

____ ____________________
Signature of Person Conducting the Assent Discussion Date

____
Printed Name of Person Conducting the Assent Discussion

Hôpitaux Shriners pour enfants® Shriners Hospitals for Children® Canada

Shriners Hospitals for Children®-Canada

INFORMED CONSENT AND AUTHORIZATION TO TAKE PART IN A RESEARCH PROJECT OR STUDY (DYAD INTERVIEWS)

**TITLE:**	Usability Testing of an E-Health Program for Youth with Osteogenesis Imperfecta (Phase II)
**SHRINERS PROTOCOL NO.:**	CAN2105
**IRB APPROVAL NO.:**	*TBD*
**PRINCIPAL INVESTIGATOR:**	Argerie Tsimicalis, RN PhD<br>1003 Décarie Blvd. Suite 2.19<br>Montreal, Quebec, H4A IM0<br>Canada<br>(514) 842-4464 ext. 2268<br>atsimicalis@shrinenet.org
**CLINICAL RESEARCH COORDINATOR:**	Sofia Addab, MSc<br>1003 Décarie Blvd. Suite 2.19<br>Montreal, Quebec, H4A IM0<br>Canada<br>(514) 842-4464 ext. 2264<br>sofia.addab@shrinenet.org

PARTICIPANT: ____

When we say "you" in this informed consent form, we mean you or your child; "we" means the doctors and research staff.

SUMMARY

You are being asked to take part in a research study. The purpose of this informed consent form is to help you decide if you want to be in the research study.

Research studies include only people who want to take part. Before you decide if you want to take part, it is important that you read and understand this consent form. Please take your time to make your decision. Discuss it with your parents, friends, and family. We encourage you to include your parents in the discussion and decision. Please ask questions about anything that you do not understand before deciding whether or not to participate.

The person in charge of this study is Dr. Argerie Tsimicalis. There may be other people on the research team helping during the study.

Things to know and understand before deciding to take part in a research study:

- The main goal of a research study is to learn things to help patients in the future.
- The decision to join or not join the research study will not cause you to lose any medical benefits. If you decide not to take part in this study, your doctor will continue to treat you.
- After reading the consent form and having a discussion with the research staff, you should know which parts of the study are experimental and which are standard medical care.

Why are you being asked to take part in this study?

You are being asked to take part in a study because you are a teen, or you care for a teen who has been diagnosed with OI and will be in the future transitioning to the adult health care system.

The purpose of this study is to gather your input on the usability of a program called "*Teens Taking Charge: Managing OI Online" (Teens OI).* This is an interactive, e-health program designed to help youth learn about their OI, manage their condition, and offer social support. There is also a section for parents.

The information you provide us will help develop an effective, user-friendly, Internet-based, self-management program for youth with OI to help them better cope with OI, improve their health-related quality of life, and enhance their readiness to transition to adult health care. It will also help parents/caregivers prepare for the transition.

How many people will take part in the study?

About 32 adolescents and 32 parents will participate in the study to test the usability of the developed *Teens OI* website.

An additional 8-12 individuals (patients, young adults with OI, parents, healthcare professionals, and decision-makers) will also participate in the study. They will form the Council, which will help oversee the usability testing of the *Teens OI* website.

What will happen in this study?

If you can be in the study, and you choose to take part, you will be asked to complete a sociodemographic questionnaire on Qualtrics, which will ask you questions about your health and internet use. Qualtrics is a Shriners-approved and secure platform for the design and administration of surveys, often used for research purposes. This will take about 15 minutes. We will explain to you how to access the questionnaires online and how to complete them before your interview. We will also send you reminders. If you are unable to complete the questionnaire online, we can send it to you by mail/email or do it with you over the phone or during the interview.

Then, you will participate in an interview to test the usability of the developed *Teens OI* website. The interviewer will ask you to perform specific tasks on the website and will record the time spent to complete the task, the accuracy of the task, and the completeness of the task. For

example, the interviewer may say, "You are a youth with OI entering your last year of high school and getting ready to apply to university. Using the website, find information regarding university applications in the context of OI." You will be encouraged to think aloud as you search the website for the information, including the challenges you are experiencing, as you are navigating the website. At the end of the usability testing, you will complete the System Usability Scale, a satisfaction questionnaire. Overall, the usability testing will last 45-60 minutes.

The interview may be done in-person or online using the Microsoft Teams platform. If you choose to do the interview online, we will schedule a time and date, and send you the link via the email you provide to us. The interview will be audio-recorded, and your feedback about the website will be noted. You may choose to have the interview as a youth/parent dyad, or separately.

Finally, you will be invited to a final symposium to gather your last feedback about the *Teens OI* website.

What will happen in this study that is "research"?

There are no experimental tests used in this study. Your prospective participation in the interview is part of the research study.

How long does the study last?

The socio-demographic questionnaire should take no longer than 5-10 minutes. Following the completion of the questionnaire, there will be one 45-60 minute interview, including the completion of the System Usability Scale. The final symposium, developed with input from the Council, may be for a few hours to a whole day.

Overall, the study will take place over a 9-12 month period.

Can you stop being in the study?

Yes. You can drop out of the study at any time and no one will be upset. It will not affect your other care and treatment received at Shriners Hospitals for Children. You do this by contacting the study coordinator, Sofia Addab at 514-842-4664 ext 2264 or by email at sofia.addab@shrinenet.org. It is important to know that there will be no negative impact if you choose to stop.

If you drop out of the study, no new health information identifying you will be gathered after that date. Information that has already been gathered may still be used and given to others. Additionally, if you choose to participate but do not complete study procedures, you may be dropped from the study.

The researcher conducting this study may decide to take you off this study without your consent under the following circumstances:

- If she or he believes that it is in your best interest
- If study procedures are not followed or if you do not show up for the interview

What are the risks of the study?

This study involves no more than minimal risk, which means that we expect that you will have no more risk than those you have in your normal daily life. You are free to decline to answer any question that makes you uncomfortable.

Since some parts of the study will be carried out using an internet connection, please make sure to delete your cookies and close your internet browser after completing the survey to maximize privacy and data protection.

Are there benefits to taking part in the study?

You will not directly benefit from participating in this study. Your feedback will be used to inform the usability of *Teens OI*, which may help other adolescents to better manage their OI. If you would like to provide your contact information (email or mailing address), we will send you information on the study findings and updates on the online self-management program.

What other options are available to you?

This is not a treatment study. Your alternative is not to be in this study.

What are the costs of taking part in this study?

You will not be paid for being in this study.

In the case of injury or illness resulting from this study, medical treatment is available. Please notify the investigator as soon as possible if you need to seek medical care for an injury or illness resulting from this study. Although Shriners Hospitals for Children is not able to offer financial compensation for an injury or illness resulting from this study, to the extent Shriners Hospitals for Children provides the needed medical treatment at its facility, that treatment may be provided at no cost to you. If you obtain that care somewhere else, your usual healthcare coverage may apply. Although Shriners Hospitals for Children has not set aside any funds to pay you for injury or illness, you do not give up any of your legal rights by signing this form.

What are your rights if you take part in this study?

Voluntary Participation: Taking part in this study is your choice. You may choose not to take part in the study or you may leave at any time. Your decision about being in this study or refusal will not affect your care at Shriners Hospitals for Children. If you have any questions about the study, you can always talk to one of the study staff. Do not agree to take part in the study unless you have had the chance to ask questions and have received clear answers.

New Findings: We do not expect to find any new information that may affect your willingness to stay in this study during your brief involvement. However, we will tell you if there were any new findings.

Who will be paying for the study?

Shriners Hospitals for Children, the sponsor, will pay for this study.

Whom can you call if you have questions or problems?

If you have any questions, please ask us. If you have any questions later or in the event of research-related injury, please contact Dr. Argerie Tsimicalis by telephone at 514-842-4464 ext. 2268 or by email at atsimicalis@shrinenet.org.

You can contact the Ethics Officer for the McGill IRB, Ms. Ilde Lepore, by telephone at (514) 398-8302 or by email at ilde.lepore@mcgill.ca for answers to questions you might have about research and your rights as a research participant.

It is important that you tell your study doctor if you feel that you have been injured because of taking part in this study. In the event of research-related injury, please call Ms. Sofia Addab at (514) 842-4464 ext. 2264

AUTHORIZATION TO USE AND DISCLOSE INFORMATION FOR RESEARCH PURPOSES

What information may be used and given to others?

The research team may use your personal and health information, using only the minimal health information needed to complete the study.

Personal health information (PHI) obtained from the following records may be used or disclosed:

- Electronic medical record or sociodemographic questionnaire: date of birth/age, sex, race/ethnicity, diagnosis, and contact information

Who may use and give out information about you in connection with this research study?

- The principal investigator, Dr. Argerie Tsimicalis
- Other members of the research team affiliated with Shriners Hospitals for Children who might need to use or disclose information from your study file in connection with this study.

Who might get this information?

The people mentioned above may share or disclose information from your study file with the following entities or people in connection with a research study.

- Shriners Hospitals for Children, the sponsor of this research. “Sponsor” means any persons or companies that are working for or with Shriners Hospitals for Children
- The McGill Faculty of Medicine Institutional Review Board
- Regulatory authorities such as Health Canada

How will information about you be kept private?

You will not be identified by name or other personal identification. To protect your identity, the research team will only use code numbers to refer to your information. A list linking the code number with your name will be stored in a separate file. All data, with identifying information removed, will be entered and securely stored into a data management system.

We will do our best to make sure that the personal information in your study file will be kept private. However, absolute confidentiality cannot be guaranteed because of the need to give information to the above-mentioned parties. Your personal information may also be given out if required by law, such as pursuant to a court order.

Data will be kept for seven years following the publication of the study results. Subsequently, paper documents will be shredded, and electronic data will be destroyed by secure deletion according to the Retention and Destruction policy at Shriners Hospital for Children.

Why will this information be used and/or given to others?

- To do the research in this study,
- To examine results related to this study, and
- To see if this study was done right.

The results of this research study may be presented at meetings or in publications. Your name and other personal information will not be used in those presentations.

What if you decide not to give permission to use and give out your health information?

Then you may still be able to be in this research study, but the results may be less useful for this research.

May you review or copy your information?

Yes, but only after the research is over.

May you withdraw or revoke (cancel) your permission to use and give out your health information?

This permission will be good until December 31, 2070.

You may withdraw or take away your permission to use and disclose your health information at any time. You do this by sending a written notice to the study doctor. If you withdraw your permission, you will not be able to stay in this study.

When you withdraw your permission, no new health information identifying you will be gathered after that date. Information that has already been gathered may still be used and given to others.

Is your health information protected after it has been given to others?

There is a risk that your information may be given to others without your permission. Any information that is shared may no longer be protected by federal privacy rules.

Title of Research Project: Usability Testing of an E-Health Program for Youth with Osteogenesis Imperfecta (Phase II)

Your signature below will show that you acknowledge and agree with the following statements:

- **I have read this consent form (or it has been read to me)**
- **I will freely take part in this research study,**
- **I have had a chance to ask questions and all of my questions have been answered**
- **I authorize the release of medical and research records for the purpose of this study**
- **I have read and understood the information above**
- **By signing this consent form, I have not given up any of my legal rights.**

You will be given a signed and dated copy of this informed consent form, which is yours to keep.

CONSENT SIGNATURE:

____ __________
Signature of Participant Date

____
Email address

....

Using language that is understandable and appropriate, I have discussed this project and the items listed above with the participant and/or his parent/legal guardian.

____ ____________________
Signature of the person who conducted the informed consent discussion Date

____ ____________________
Print Name Study Role

ASSENT SECTION:

Statement of person conducting assent discussion:

- I have explained all aspects of the research to the subject to the best of his or her ability to understand.
- I have answered all the questions of the subject relating to this research.
- I believe the subject's decision to enroll is voluntary.
- The study staff agrees to respect the subject's physical or emotional dissent at any time during this research when that dissent pertains to anything being done solely for the purpose of this research.

a. I have explained the study to the extent compatible with the subject's capability, and the subject has agreed to be in the study.

OR

b. The subject is not able to assent because the capability of the subject is so limited that the subject cannot reasonably be consulted.

____________________________________ __________________

Signature of Person Conducting the Assent Discussion Date

____

Printed Name of Person Conducting the Assent Discussion

Statement of Parent or Guardian:

My child/the subject appears to understand the research to the best of his or her ability and has agreed to participate.

____________________________________ __________________

Signature of Parent or Guardian Date

Shriners Hospitals for Children®-Canada

INFORMED CONSENT AND AUTHORIZATION
TO TAKE PART IN A RESEARCH PROJECT OR STUDY
(COUNCIL: HEALTHCARE PROFESSIONALS AND DECISION MAKERS)

TITLE: Design and Development of an E-health Program for Youth with Osteogenesis Imperfecta (Phase I)

SHRINERS PROTOCOL NO.: CAN2101
IRB APPROVAL NUMBER: A04-B31-21B

INVESTIGATOR: Argerie Tsimicalis, RN, PhD
1003 Decarie Blvd
Montreal, Quebec, H4A IM0
Canada

STUDY-RELATED PHONE NUMBER(S): Argerie Tsimicalis, RN PhD
(514) 842-4464 ext. 2268

PARTICIPANT: ___________________________________

SUMMARY

Research studies include only people who want to take part. The purpose of this informed consent information sheet is to help you decide if you want to be in the research study.

Before you decide if you want to take part, it is important that you read and understand this consent form. Please take your time to make your decision. Please ask questions about anything that you do not understand before deciding whether to participate.

The person in charge of this study is Argerie Tsimicalis, RN PhD. There may be other people on the research team helping during the study.

Why are you being asked to take part in this study?

You are being asked to take part in this research study because you deliver care to patients with OI or are a decision maker involved in the care of patients with OI at the Shriners Hospitals for Children®-Canada.

The purpose of the study is to design and develop a program called "*Teens Taking Charge: Managing OI Online" (Teens OI).* This is an interactive, e-health program designed to help youth learn about their OI, manage their condition, and offer social support. There is also a section for parents.

The information you provide us as a member of the Council will help develop an effective Internet-based self- management program for youth with OI, to help them better cope with OI, improve their health-related quality of life and enhance their readiness to transition to adult health care. It will also help parents prepare for their youth's transition.

How many people will take part in the study?

The Council will consist of 8-12 individuals, which may include patients, young adults with OI, parents, health care professionals, or decision makers.

An additional 20 youth with OI and 20 parents will participate in the study and help as well in the design and development of the website.

What will happen in this study?

If you can be in the study, and you choose to take part, you will be part of a Council that will oversee the development and design of the Teens OI website. You will participate in four focus groups (1-hour each, for a total of 4 hours) with other members of the Council. The focus groups may be done in-person or online using the Microsoft Teams platform. In the event the focus groups will be online, we will schedule a time and date, and send you the link via the email you provide to us. With your permission, we would like to audio-record the focus group discussions.

In the first focus group, you will receive training in patient engagement and partnerships, brainstorm content for the website, and identify OI experts that should be invited to the final symposium.

During the second and third focus groups, you will review feedback received from youth with OI and their parents on the content and various features of the website. You will contribute to the design and development of the website as well. You will provide feedback on the preparation for the Symposium, and be invited to attend the event.

During the last focus group, you will be invited to finalize the website based on the findings from the Symposium, and approve any final changes allowing the research team to proceed to the next phase of study.

What will happen in this study that is "research"?

There are no experimental tests used in this study.

How long does the study last?

Each focus group will last about 1 hour, for a total of 4 hours. The final Symposium will be developed with input from the Council. This event may be for a few hours to a whole day. Overall, the study will take place over a 9-12 month period.

Can you stop being in the study?

Yes. You can drop out of the study at any time. No one will be upset. It will not affect your employment at the hospital. You can do this by telling one of the members of the research team.

There are no anticipated circumstances under which we would decide to take you off the study.

What are the risks of the study?

This study involves no more than minimal risks, which means that we expect that you will have no more risk than those you have in your normal daily life.

Are there benefits to taking part in the study?

You will not directly benefit from participating in this study. Your feedback will be used to inform the design and development of the online self-management program for adolescents with OI, which may help other adolescents to better manage their OI. If you would like to provide your contact information (email or mailing address) we will send you information on the study findings and updates on the online self- management program.

What other options are available to you?

If you decide not to enter this study, your alternative is not to be in this study.

How will information about you be kept private?

You will not be identified by name. To protect your identity, the research team will only use code numbers to refer to your information. The code numbers will be stored in a separate file. Transcriptions of the focus groups will be modified to exclude any information that may identify you. The consent forms will be stored in a locked cabinet in the PI's office at the hospital. This data will be entered into an encrypted Excel file on a password-protected computer. Any audio recordings and electronic materials will be stored on a password-protected computer in the PI's office. Access to the filing cabinet and the computer will only be available to the research team.

If information from this study is published or presented at scientific meetings, your name and other personal information will not be used.

Your personal information may be given out if required by law. Shriners Hospital for Children Clinical Research Monitors, the McGill Institutional Review Board, employees of the funder or sponsor, or the regulator of the study may see your personal information to check on the study. By signing this consent form, you agree to let these people look at your records. We will give you a copy as well.

Data will be kept for seven years following publication of the study results. Subsequently, paper documents will be shredded and electronic data will be destroyed by secure delete according to the Retention and Destruction policy at Shriners Hospital for Children.

What are the costs?

You will not be paid for being in this study.

There are no costs to take part in this study. You will receive a personalized certificate of appreciation.

What are your rights if you take part in this study?

Voluntary Participation: Taking part in this study is your choice. You may choose not to take part in the study or you may leave at any time. Your decision about being in this study will not affect your employment at Shriners Hospitals for Children®-Canada. If you have any questions about the study, you can always talk to one of the study staff. Do not sign this form unless you have had the chance to ask questions and have received clear answers.

New Findings: We do not expect to find any new information that may affect your willingness to stay in this study during your brief involvement. However, we will tell you if there were any new findings.

Whom can you call if you have questions or problems?

If you have any questions, please ask us. If you have any questions later, please contact Dr. Argerie Tsimicalis by telephone at (514) 842-4464 ext. 2268 or by email at: atsimicalis@shrinenet.org. You can contact the Ethics Officer for the McGill IRB, Ms. Ilde Lepore, by telephone at (514) 398-8302 or by email at ilde.lepore@mcgill.ca for answers to questions you might have about research and about your rights as a research participant.

Title of Research Project: Design and Development of an E-health Program for Youth with Osteogenesis Imperfecta (Phase I)

Your signature below will show that you acknowledge and agree that:

- **You have read this consent form (or it has been read to me)**
- **You will freely take part in this research study,**
- **You have had a chance to ask questions and all of your questions have been answered**
- **You authorize the release of medical and research records for the purpose of this study**
- **You have read and understood the information above**
- **By signing this consent form, you have not given up any of my legal rights.**

You will be given a signed and dated copy of this informed consent form which is yours to keep.

<u>CONSENT SIGNATURE:</u>

_______________________________________ __________

Signature of Participant Date

In the event of an online focus group, email: _______________________________________

....

Using language that is understandable and appropriate, I have discussed this project and the items listed above with the participant and/or his parent/legal guardian.

____ ____________________

Signature of the person who conducted the informed consent discussion Date

____ ____________________

____ ____________________

Print Name Study Role

Shriners Hospitals for Children®-Canada

INFORMED CONSENT AND AUTHORIZATION
TO TAKE PART IN A RESEARCH PROJECT OR STUDY
(COUNCIL: YOUTH, YOUNG ADULTS WITH OI, AND PARENTS)

TITLE: Design and Development of an E-health Program for Youth with Osteogenesis Imperfecta (Phase I)

SHRINERS PROTOCOL NO.: CAN2101
IRB APPROVAL NUMBER: A04-B31-21B

INVESTIGATOR: Argerie Tsimicalis, RN, PhD
1003 Decarie Blvd
Montreal, Quebec, H4A IM0
Canada

STUDY-RELATED PHONE NUMBER(S): Argerie Tsimicalis, RN PhD
(514) 842-4464 ext. 2268

PARTICIPANT: ____

When we say "you" in this informed consent information sheet, we mean you or you and your child; "we" means the doctors and other research staff.

SUMMARY

You are being asked to take part in a research study. The purpose of this informed consent information sheet is to help you decide if you want to be in the research study.

Research studies include only people who want to take part. Before you decide if you want to take part, it is important that you read and understand this consent form. Please take your time to make your decision. Discuss it with your parents, friends and family. We encourage you to include your parents in the discussion and decision. Please ask questions about anything that you do not understand before deciding whether or not to participate.

The person in charge of this study is Dr. Argerie Tsimicalis. There may be other people on the research team helping during the study.

Things to know and understand before deciding to take part in a research study:

- The main goal of a research study is to learn things to help patients in the future.
- The main goal of regular medical care is to help each patient.
- The decision to join or not join the research study will not cause you to lose any

medical benefits. If you decide not to take part in this study, your doctor will continue to treat you.

- After reading the consent form and having a discussion with the research staff, you should know which parts of the study are experimental and which are standard medical care.

Why are you being asked to take part in this study?

You are being asked to take part in a study because you are a teen who has been diagnosed with OI and will be in the future transitioning to the adult health care system, or are a young adult with OI, or are the parent of a teen with OI.

The purpose of this study is to gather your input as a member of the Council into the design and development of a program called "*Teens Taking Charge: Managing OI Online" (Teens OI).* This is an interactive, e-health program designed to help youth learn about their OI, manage their condition, and offer social support. There is also a section for parents.

The information you provide us will help develop an effective Internet-based self- management program for youth with OI, like you, to help them better cope with OI, improve their health-related quality of life and enhance their readiness to transition to adult health care. It will also help parents prepare for their child's transition.

How many people will take part in the study?

The Council will consist of 8-12 individuals, which may include patients, young adults with OI, parents, health care professionals, or decision makers.

An additional 20 youth with OI and 20 parents will participate in the study and help as well in the design and development of the website.

What will happen in this study?

If you can be in the study, and you choose to take part, you will be part of a Council that will oversee the design and development of the Teens OI website. You will participate in four focus groups (1-hour each, for a total of 4 hours) with other members of the Council. The focus groups may be done in-person or online using the Microsoft Teams platform. In the event the focus groups will be online, we will schedule a time and date, and send you the link via the email you provide to us. With your permission, we would like to audio-record the focus group discussions.

In the first focus group, you will receive training in patient engagement and partnerships, brainstorm content for the website, and identify OI experts that should be invited to the final symposium.

During the second and third focus groups, you will review feedback received from youth with OI and their parents on the content and various features of the website. You will contribute to the design and development of the website as well. You will provide feedback on the preparation for the Symposium, and be invited to attend the event.

During the last focus group, you will be invited to finalize the website based on the findings from the Symposium, and approve any final changes allowing the research team to proceed to the next phase of study.

What will happen in this study that is "research"?

There are no experimental tests used in this study.

How long does the study last?

Each focus group will last about 1 hour, for a total of 4 hours. The final Symposium will be developed with input from the Council. This event may be for a few hours to a whole day. You may decide at a later point if you would like to attend the Symposium. Overall, the study will take place over a 9-12 month period.

Can you stop being in the study?

Yes. You can drop out of the study at any time and no one will be upset. It will not affect your other care and treatment received at Shriners Hospitals for Children. You do this by contacting the study coordinator, Sofia Addab at 514-842-4664 ext 2264 or by email at sofia.addab@shrinenet.org.

It is important to tell a member of the research team if you are thinking about stopping so the impact, if any, from stopping the study can be discussed.

When you drop out of the study, no new health information identifying you will be gathered after that date. Information that has already been gathered may still be used and given to others. Additionally, if you choose to participate but do not complete study procedures, you may be dropped from the study.

The researcher conducting this study may decide to take you off this study without your consent under the following circumstances:

- If she or he believes that it is in your best interest
- If study procedures are not followed or if visits are not kept

What are the risks of the study?

This study involves no more than minimal risks, which means that we expect that you will have no more risk than those you have in your normal daily life or routine physical (*or psychological*) examination or tests.

Are there benefits to taking part in the study?

You will not directly benefit from participating in this study. Your feedback will be used to inform the design of the online self-management program for adolescents with OI, which may help other adolescents to better manage their OI. If you would like to provide your contact information (email or mailing address) we will send you information on the study findings and updates on the online self- management program.

What other options are available to you?

This is not a treatment study. Your alternative is not to be in this study.

What are the costs to taking part in this study?

You will not be paid for being in this study.

There are no costs to take part in this study. You will receive a personalized certificate of appreciation documenting your volunteer hours.

In the case of injury or illness resulting from this study, medical treatment is available. To the extent the Shriners Hospitals for Children provides medical services at its facility, those will be at no cost. If you get that care somewhere else, your usual healthcare coverage would apply. Shriners Hospitals for Children is not able to offer financial compensation for a research- related injury or other problems. Although no funds have been set aside to pay you for injury or illness, you do not give up any of your legal rights by signing this form.

What are your rights if you take part in this study?

Voluntary Participation: Taking part in this study is your choice. You may choose not to take part in the study or you may leave at any time. Your decision about being in this study or refusal will not affect your care at Shriners Hospitals for Children. If you have any questions about the study, you can always talk to one of the study staff. Do not agree to take part in the study unless you have had the chance to ask questions and have received clear answers.

New Findings: We do not expect to find any new information that may affect your willingness to stay in this study during your brief involvement. However, we will tell you if there were any new findings.

Who will be paying for the study?

Shriners Hospitals for Children, the sponsor, will pay for this study.

Whom can you call if you have questions or problems?

If you have any questions, please ask us. If you have any questions later or in the event of research-related injury, please contact Dr. Argerie Tsimicalis by telephone at 514-842-4464 ext. 2268 or by email at: atsimicalis@shrinenet.org. You can contact the Ethics Officer for the McGill IRB, Ms. Ilde Lepore, by telephone at (514) 398-8302 or by email at ilde.lepore@mcgill.ca for answers to questions you might have about research and about your rights as a research participant.

It is important that you tell your study doctor if you feel that you have been injured because of taking part in this study. In the event of research-related injury, please call Ms. Sofia Addab at (514) 842-4464 ext. 2264

AUTHORIZATION TO USE AND DISCLOSE INFORMATION FOR RESEARCH PURPOSES

What information may be used and given to others?

The research team may use your personal and health information, using only the minimal heath information needed to complete study.

Personal health information obtained from the following records may be used or disclosed:

- date of birth, gender, race/ethnicity, and diagnosis

Who may use and give out information about you in connection with this research study?

- The principal investigator, Dr. Argerie Tsimicalis
- Other members of the research team affiliated with Shriners Hospitals for Children who might need to use or disclose information from your study file in connection with this study.

Who might get this information?

The people mentioned above may share or disclose information from your study file with the following entities or people in connection with a research study.

- Shriners Hospitals for Children, the sponsor of this research. “Sponsor” means any persons or companies that are working for or with Shriners Hospitals for Children
- The McGill Faculty of Medicine Institutional Review Board
- Regulatory authorities such as Health Canada

How will information about you be kept private?

You will be assigned a unique identification code, which will be linked to your study materials. Your code will be kept separate from your study data and consent forms and then stored in an encrypted database on a password-protected computer in a locked office. Your completed questionnaires will be stored in a locked cabinet in a locked office and will be accessible only to the research team. The information from the questionnaires will be entered into an encrypted database saved on the server, which will be accessed from a password- protected computer. Digital interview audio recordings and transcripts will be coded and de-identified from the individual interviews and will be stored in an encrypted database saved on the server accessed from a password-protected computer. If information from this study is published or presented at scientific meetings, your name and other personal information will not be used.

Data will be kept for seven years following publication of the study results. Subsequently, paper documents will be shredded and electronic data will be destroyed by secure delete according to the Retention and Destruction policy at Shriners Hospital for Children.

Why will this information be used and/or given to others?

- To do the research in this study,
- To examine results related to this study, and
- To see if this study was done right

The results of this research study may be presented at meetings or in publications. Your name and other personal information will not be used in those presentations.

What if you decide not to give permission to use and give out my health information?

Then you may still be able to be in this research study, but the results may be less useful for this research.

May you review or copy your information?

Yes, but only after the research is over.

May you withdraw or revoke (cancel) your permission to use and give out your health information?

This permission will be good until December 31, 2070.

You may withdraw or take away your permission to use and disclose your health information at any time. You do this by sending a written notice to the study doctor. If you withdraw your permission, you will not be able to stay in this study.

When you withdraw your permission, no new health information identifying you will be gathered after that date. Information that has already been gathered may still be used and given to others.

Is your health information protected after it has been given to others?

There is a risk that your information may be given to others without your permission. Any information that is shared may no longer be protected by federal privacy rules.

Title of Research Project: Design and Development of an E-health Program for Youth with Osteogenesis Imperfecta (Phase I)

Your signature below will show that you acknowledge and agree with the following statements:

- **I have read this consent form (or it has been read to me)**
- **I will freely take part in this research study,**
- **I have had a chance to ask questions and all of my questions have been answered**
- **I authorize the release of medical and research records for the purpose of this study**
- **I have read and understood the information above**
- **By signing this consent form, I have not given up any of my legal rights.**

You will be given a signed and dated copy of this informed consent form which is yours to keep.

CONSENT SIGNATURE:

____ __________

Signature of Participant Date

OR

_______________________________________ __________ ____________________

Signature of Parent or Subject's Legally Authorized Representative (Parent, Legal Guardian, etc.) Date Relationship to Participant

In the event of an online focus group, email: ____

....

Using language that is understandable and appropriate, I have discussed this project and the items listed above with the participant and/or his parent/legal guardian.

____ _____________________

Signature of the person who conducted the informed consent discussion Date

____ _____________________

Print Name Study Role

ASSENT SECTION:

Statement of person conducting assent discussion:

- I have explained all aspects of the research to the subject to the best of his or her ability to understand.
- I have answered all the questions of the subject relating to this research.
- The subject agrees to be in the research.
- I believe the subject's decision to enroll is voluntary.
- The study doctor and study staff agrees to respect the subject's physical or emotional dissent at any time during this research when that dissent pertains to anything being done solely for the purpose of this research.

Statement of Parent or Guardian:

My child appears to understand the research to the best of his or her ability and has agreed to participate.

____ __________________
Signature of Parent or Guardian Date

❑ I have explained the study to the extent compatible with the subject's capability, and the subject has agreed to be in the study.

OR

❑ The subject is not able to assent because the capability of the subject is so limited that the subject cannot reasonably be consulted.

______________________________________ __________________
Signature of Person Conducting the Assent Discussion Date

____
Printed Name of Person Conducting the Assent Discussion

Shriners Hospitals for Children®-Canada

INFORMED CONSENT AND AUTHORIZATION TO TAKE PART IN A RESEARCH PROJECT OR STUDY (AGES 14+)

TITLE: Design and Development of an E-health Program for Youth with Osteogenesis Imperfecta (Phase I)

SHRINERS PROTOCOL NO.: CAN2101
IRB APPROVAL NUMBER: A04-B31-21B

INVESTIGATOR: Argerie Tsimicalis, RN, PhD
1003 Decarie Blvd
Montreal, Quebec, H4A IM0
Canada

STUDY-RELATED PHONE NUMBER(S): Argerie Tsimicalis, RN PhD
(514) 842-4464 ext. 2268

PARTICIPANT: ____

When we say “you” in this informed consent information sheet, we mean you or you and your child; “we” means the doctors and other research staff.

SUMMARY

You are being asked to take part in a research study. The purpose of this informed consent information sheet is to help you decide if you want to be in the research study.

Research studies include only people who want to take part. Before you decide if you want to take part, it is important that you read and understand this consent form. Please take your time to make your decision. Discuss it with your parents, friends and family. We encourage you to include your parents in the discussion and decision. Please ask questions about anything that you do not understand before deciding whether or not to participate.

The person in charge of this study is Dr. Argerie Tsimicalis. There may be other people on the research team helping during the study.

Things to know and understand before deciding to take part in a research study:

- The main goal of a research study is to learn things to help patients in the future.
- The main goal of regular medical care is to help each patient.
- The decision to join or not join the research study will not cause you to lose any

medical benefits. If you decide not to take part in this study, your doctor will continue to treat you.

- After reading the consent form and having a discussion with the research staff, you should know which parts of the study are experimental and which are standard medical care.

Why are you being asked to take part in this study?

You are being asked to take part in a study because you are a teen who has been diagnosed with OI and will be in the future transitioning to the adult health care system.

The purpose of this study is to gather your input into the design and development of a program called "*Teens Taking Charge: Managing OI Online" (Teens OI).* This is an interactive, e-health program designed to help youth learn about their OI, manage their condition, and offer social support. There is also a section for parents.

The information you provide us will help develop an effective Internet-based self- management program for youth with OI- like you, to help them better cope with OI, improve their health-related quality of life and enhance their readiness to transition to adult health care. It will also help your parent(s) prepare for your transition.

How many people will take part in the study?

About 20 adolescents and 20 parents will participate in the study.

An additional 8-12 individuals (patients, young adults with OI, parents, health care professionals, and decision makers) will also participate in the study. They will form the Council, which will help oversee the design and development of the Teens OI website.

What will happen in this study?

If you can be in the study, and you choose to take part, you will start by completing a socio-demographic questionnaire, which will ask you questions about your health and internet-use. This will take about 10 minutes.

Then, you will participate in an interview. The interviewer will ask you about how you manage your OI, will ask you to review sections of the program being developed and will offer you feedback, and inquire whether this online program would be useful for you to help manage your OI. The interview will last 45-60 minutes.

The interview may be done in-person or online using the Microsoft Teams platform. If you choose to do the interview online, we will schedule a time and date, and send you the link via the email you provide to us. The interview will be audio-recorded, and your feedback about the program being developed will be noted. You may choose to have the interview with your parent/legal-guardian, or separately.

Finally, you will be invited to a final symposium to gather your last feedback about the Teens OI website.

What will happen in this study that is "research"?

There are no experimental tests used in this study.

How long does the study last?

The socio-demographic questionnaire should take no longer than 5-10 minutes. Following the completion of the questionnaire, there will be one 45-60 minute interview. The final Symposium will be developed with input from the Council. This event may be for a few hours to a whole day.

Overall, the study will take place over a 9-12 month period.

Can you stop being in the study?

Yes. You can drop out of the study at any time and no one will be upset. It will not affect your other care and treatment received at Shriners Hospitals for Children. You do this by contacting the study coordinator, Sofia Addab at 514-842-4664 ext 2264 or by email at sofia.addab@shrinenet.org.

It is important to tell a member of the research team if you are thinking about stopping so the impact, if any, from stopping the study can be discussed.

When you drop out of the study, no new health information identifying you will be gathered after that date. Information that has already been gathered may still be used and given to others. Additionally, if you choose to participate but do not complete study procedures, you may be dropped from the study.

The researcher conducting this study may decide to take you off this study without your consent under the following circumstances:

- If she or he believes that it is in your best interest
- If study procedures are not followed or if visits are not kept

What are the risks of the study?

You will be asked to speak about your experience living with OI and provide input on a program designed for individuals like you. This task may cause you to become uncomfortable. You are free to decline to answer any question that makes you uncomfortable. You will be able to stop the interview at any time.

Are there benefits to taking part in the study?

You will not directly benefit from participating in this study. Your feedback will be used to inform the design of the online self-management program for adolescents with OI, which may help other adolescents to better manage their OI. If you would like to provide your contact information (email or mailing address) we will send you information on the study findings and updates on the online self- management program.

What other options are available to you?

This is not a treatment study. Your alternative is not to be in this study.

What are the costs to taking part in this study?

You will not be paid for being in this study.

There are no costs to take part in this study. You will receive a personalized certificate of appreciation documenting your volunteer hours.

In the case of injury or illness resulting from this study, medical treatment is available. To the extent the Shriners Hospitals for Children provides medical services at its facility, those will be at no cost. If you get that care somewhere else, your usual healthcare coverage would apply. Shriners Hospitals for Children is not able to offer financial compensation for a research- related injury or other problems. Although no funds have been set aside to pay you for injury or illness, you do not give up any of your legal rights by signing this form.

What are your rights if you take part in this study?

Voluntary Participation: Taking part in this study is your choice. You may choose not to take part in the study or you may leave at any time. Your decision about being in this study or refusal will not affect your care at Shriners Hospitals for Children. If you have any questions about the study, you can always talk to one of the study staff. Do not agree to take part in the study unless you have had the chance to ask questions and have received clear answers.

New Findings: We do not expect to find any new information that may affect your willingness to stay in this study during your brief involvement. However, we will tell you if there were any new findings.

Who will be paying for the study?

Shriners Hospitals for Children, the sponsor, will pay for this study.

Whom can you call if you have questions or problems?

If you have any questions, please ask us. If you have any questions later or in the event of research-related injury, please contact Dr. Argerie Tsimicalis by telephone at 514-842-4464 ext. 2268 or by email at: atsimicalis@shrinenet.org. You can contact the Ethics Officer for the McGill IRB, Ms. Ilde Lepore, by telephone at (514) 398-8302 or by email at ilde.lepore@mcgill.ca for answers to questions you might have about research and about your rights as a research participant.

It is important that you tell your study doctor if you feel that you have been injured because of taking part in this study. In the event of research-related injury, please call Ms. Sofia Addab at (514) 842-4464 ext. 2264

AUTHORIZATION TO USE AND DISCLOSE INFORMATION FOR RESEARCH PURPOSES

What information may be used and given to others?

The research team may use your personal and health information, using only the minimal heath information needed to complete study.

Personal health information obtained from the following records may be used or disclosed:

- date of birth, gender, race/ethnicity, and diagnosis

Who may use and give out information about you in connection with this research study?

- The principal investigator, Dr. Argerie Tsimicalis
- Other members of the research team affiliated with Shriners Hospitals for Children who might need to use or disclose information from your study file in connection with this study.

Who might get this information?

The people mentioned above may share or disclose information from your study file with the following entities or people in connection with a research study.

- Shriners Hospitals for Children, the sponsor of this research. “Sponsor” means any persons or companies that are working for or with Shriners Hospitals for Children
- The McGill Faculty of Medicine Institutional Review Board
- Regulatory authorities such as Health Canada

How will information about you be kept private?

You will be assigned a unique identification code, which will be linked to your study materials. Your code will be kept separate from your study data and consent forms and then stored in an encrypted database on a password-protected computer in a locked office. Your completed questionnaires will be stored in a locked cabinet in a locked office and will be accessible only to the research team. The information from the questionnaires will be entered into an encrypted database saved on the server which is accessed from a password- protected computer. Digital interview audio recordings and transcripts will be coded and de-identified from the individual interviews and will be stored in an encrypted database saved on the server which is accessed from a password-protected computer. If information from this study is published or presented at scientific meetings, your name and other personal information will not be used.

Data will be kept for seven years following publication of the study results. Subsequently, paper documents will be shredded and electronic data will be destroyed by secure delete according to the Retention and Destruction policy at Shriners Hospital for Children.

Why will this information be used and/or given to others?

- To do the research in this study,
- To examine results related to this study, and
- To see if this study was done right.

The results of this research study may be presented at meetings or in publications. Your name and other personal information will not be used in those presentations.

What if you decide not to give permission to use and give out your health information?

Then you may still be able to be in this research study, but the results may be less useful for this research.

May you review or copy your information?

Yes, but only after the research is over.

May you withdraw or revoke (cancel) your permission to use and give out your health information?

This permission will be good until December 31, 2070.

You may withdraw or take away your permission to use and disclose your health information at any time. You do this by sending a written notice to the study doctor. If you withdraw your permission, you will not be able to stay in this study.

When you withdraw your permission, no new health information identifying you will be gathered after that date. Information that has already been gathered may still be used and given to others.

Is your health information protected after it has been given to others?

There is a risk that your information may be given to others without your permission. Any information that is shared may no longer be protected by federal privacy rules.

Title of Research Project: Design and Development of an E-health Program for Youth with Osteogenesis Imperfecta (Phase I)

Your signature below will show that you acknowledge and agree with the following statements:

- **I have read this consent form (or it has been read to me)**
- **I will freely take part in this research study,**
- **I have had a chance to ask questions and all of my questions have been answered**
- **I authorize the release of medical and research records for the purpose of this study**
- **I have read and understood the information above**
- **By signing this consent form, I have not given up any of my legal rights.**

You will be given a signed and dated copy of this informed consent form which is yours to keep.

CONSENT SIGNATURE:

_______________________________________ __________

Signature of Participant Date

If choosing to do an online interview, email: _______________________________________

....

Using language that is understandable and appropriate, I have discussed this project and the items listed above with the participant and/or his parent/legal guardian.

____ ____________________

Signature of the person who conducted the informed consent discussion Date

____ ____________________

Print Name Study Role

Shriners Hospitals for Children®-Canada

INFORMED CONSENT AND AUTHORIZATION TO TAKE PART IN A RESEARCH PROJECT OR STUDY (COUNCIL: HEALTHCARE PROFESSIONALS AND DECISION MAKERS)

TITLE: Design and Development of an E-health Program for Youth with Osteogenesis Imperfecta (Phase I)

SHRINERS PROTOCOL NO.: CAN2101
IRB APPROVAL NUMBER: A04-B31-21B

INVESTIGATOR: Argerie Tsimicalis, RN, PhD
1003 Decarie Blvd
Montreal, Quebec, H4A IM0
Canada

STUDY-RELATED PHONE NUMBER(S): Argerie Tsimicalis, RN PhD
(514) 842-4464 ext. 2268

PARTICIPANT: ___________________________________

SUMMARY

Research studies include only people who want to take part. The purpose of this informed consent information sheet is to help you decide if you want to be in the research study.

Before you decide if you want to take part, it is important that you read and understand this consent form. Please take your time to make your decision. Please ask questions about anything that you do not understand before deciding whether to participate.

The person in charge of this study is Argerie Tsimicalis, RN PhD. There may be other people on the research team helping during the study.

Why are you being asked to take part in this study?

You are being asked to take part in this research study because you deliver care to patients with OI or are a decision maker involved in the care of patients with OI at the Shriners Hospitals for Children®-Canada.

The purpose of the study is to design and develop a program called "*Teens Taking Charge: Managing OI Online" (Teens OI).* This is an interactive, e-health program designed to help youth learn about their OI, manage their condition, and offer social support. There is also a section for parents.

The information you provide us as a member of the Council will help develop an effective Internet-based self- management program for youth with OI, to help them better cope with OI, improve their health-related quality of life and enhance their readiness to transition to adult health care. It will also help parents prepare for their youth's transition.

How many people will take part in the study?

The Council will consist of 8-12 individuals, which may include patients, young adults with OI, parents, health care professionals, or decision makers.

An additional 20 youth with OI and 20 parents will participate in the study and help as well in the design and development of the website.

What will happen in this study?

If you can be in the study, and you choose to take part, you will be part of a Council that will oversee the development and design of the Teens OI website. You will participate in four focus groups (1-hour each, for a total of 4 hours) with other members of the Council. The focus groups may be done in-person or online using the Microsoft Teams platform. In the event the focus groups will be online, we will schedule a time and date, and send you the link via the email you provide to us. With your permission, we would like to audio-record the focus group discussions.

In the first focus group, you will receive training in patient engagement and partnerships, brainstorm content for the website, and identify OI experts that should be invited to the final symposium.

During the second and third focus groups, you will review feedback received from youth with OI and their parents on the content and various features of the website. You will contribute to the design and development of the website as well. You will provide feedback on the preparation for the Symposium, and be invited to attend the event.

During the last focus group, you will be invited to finalize the website based on the findings from the Symposium, and approve any final changes allowing the research team to proceed to the next phase of study.

What will happen in this study that is "research"?

There are no experimental tests used in this study.

How long does the study last?

Each focus group will last about 1 hour, for a total of 4 hours. The final Symposium will be developed with input from the Council. This event may be for a few hours to a whole day. Overall, the study will take place over a 9-12 month period.

Can you stop being in the study?

Yes. You can drop out of the study at any time. No one will be upset. It will not affect your employment at the hospital. You can do this by telling one of the members of the research team.

There are no anticipated circumstances under which we would decide to take you off the study.

What are the risks of the study?

This study involves no more than minimal risks, which means that we expect that you will have no more risk than those you have in your normal daily life.

Are there benefits to taking part in the study?

You will not directly benefit from participating in this study. Your feedback will be used to inform the design and development of the online self-management program for adolescents with OI, which may help other adolescents to better manage their OI. If you would like to provide your contact information (email or mailing address) we will send you information on the study findings and updates on the online self- management program.

What other options are available to you?

If you decide not to enter this study, your alternative is not to be in this study.

How will information about you be kept private?

You will not be identified by name. To protect your identity, the research team will only use code numbers to refer to your information. The code numbers will be stored in a separate file. Transcriptions of the focus groups will be modified to exclude any information that may identify you. The consent forms will be stored in a locked cabinet in the PI's office at the hospital. This data will be entered into an encrypted Excel file on a password-protected computer. Any audio recordings and electronic materials will be stored on a password-protected computer in the PI's office. Access to the filing cabinet and the computer will only be available to the research team.

If information from this study is published or presented at scientific meetings, your name and other personal information will not be used.

Your personal information may be given out if required by law. Shriners Hospital for Children Clinical Research Monitors, the McGill Institutional Review Board, employees of the funder or sponsor, or the regulator of the study may see your personal information to check on the study. By signing this consent form, you agree to let these people look at your records. We will give you a copy as well.

Data will be kept for seven years following publication of the study results. Subsequently, paper documents will be shredded and electronic data will be destroyed by secure delete according to the Retention and Destruction policy at Shriners Hospital for Children.

What are the costs?

You will not be paid for being in this study.

There are no costs to take part in this study. You will receive a personalized certificate of appreciation.

What are your rights if you take part in this study?

Voluntary Participation: Taking part in this study is your choice. You may choose not to take part in the study or you may leave at any time. Your decision about being in this study will not affect your employment at Shriners Hospitals for Children®-Canada. If you have any questions about the study, you can always talk to one of the study staff. Do not sign this form unless you have had the chance to ask questions and have received clear answers.

New Findings: We do not expect to find any new information that may affect your willingness to stay in this study during your brief involvement. However, we will tell you if there were any new findings.

Whom can you call if you have questions or problems?

If you have any questions, please ask us. If you have any questions later, please contact Dr. Argerie Tsimicalis by telephone at (514) 842-4464 ext. 2268 or by email at: atsimicalis@shrinenet.org. You can contact the Ethics Officer for the McGill IRB, Ms. Ilde Lepore, by telephone at (514) 398-8302 or by email at ilde.lepore@mcgill.ca for answers to questions you might have about research and about your rights as a research participant.

Title of Research Project: Design and Development of an E-health Program for Youth with Osteogenesis Imperfecta (Phase I)

Your signature below will show that you acknowledge and agree that:

- **You have read this consent form (or it has been read to me)**
- **You will freely take part in this research study,**
- **You have had a chance to ask questions and all of your questions have been answered**
- **You authorize the release of medical and research records for the purpose of this study**
- **You have read and understood the information above**
- **By signing this consent form, you have not given up any of my legal rights.**

You will be given a signed and dated copy of this informed consent form which is yours to keep.

CONSENT SIGNATURE:

____ __________
Signature of Participant Date

In the event of an online focus group, email: ____

....

Using language that is understandable and appropriate, I have discussed this project and the items listed above with the participant and/or his parent/legal guardian.

____ _____________________
Signature of the person who conducted the informed consent discussion Date

____ _____________________
____ _____________________

Print Name Study Role

Shriners Hospitals for Children®-Canada

INFORMED CONSENT AND AUTHORIZATION TO TAKE PART IN A RESEARCH PROJECT OR STUDY (COUNCIL: HEALTHCARE PROFESSIONALS AND DECISION MAKERS)

TITLE: Design and Development of an E-health Program for Youth with Osteogenesis Imperfecta (Phase I)

SHRINERS PROTOCOL NO.: CAN2101
IRB APPROVAL NUMBER: A04-B31-21B

INVESTIGATOR: Argerie Tsimicalis, RN, PhD
1003 Decarie Blvd
Montreal, Quebec, H4A IM0
Canada

STUDY-RELATED PHONE NUMBER(S): Argerie Tsimicalis, RN PhD
(514) 842-4464 ext. 2268

PARTICIPANT: ___________________________________

SUMMARY

Research studies include only people who want to take part. The purpose of this informed consent information sheet is to help you decide if you want to be in the research study.

Before you decide if you want to take part, it is important that you read and understand this consent form. Please take your time to make your decision. Please ask questions about anything that you do not understand before deciding whether to participate.

The person in charge of this study is Argerie Tsimicalis, RN PhD. There may be other people on the research team helping during the study.

Why are you being asked to take part in this study?

You are being asked to take part in this research study because you deliver care to patients with OI or are a decision maker involved in the care of patients with OI at the Shriners Hospitals for Children®-Canada.

The purpose of the study is to design and develop a program called "*Teens Taking Charge: Managing OI Online" (Teens OI).* This is an interactive, e-health program designed to help youth learn about their OI, manage their condition, and offer social support. There is also a section for parents.

The information you provide us as a member of the Council will help develop an effective Internet-based self- management program for youth with OI, to help them better cope with OI, improve their health-related quality of life and enhance their readiness to transition to adult health care. It will also help parents prepare for their youth's transition.

How many people will take part in the study?

The Council will consist of 8-12 individuals, which may include patients, young adults with OI, parents, health care professionals, or decision makers.

An additional 20 youth with OI and 20 parents will participate in the study and help as well in the design and development of the website.

What will happen in this study?

If you can be in the study, and you choose to take part, you will be part of a Council that will oversee the development and design of the Teens OI website. You will participate in four focus groups (1-hour each, for a total of 4 hours) with other members of the Council. The focus groups may be done in-person or online using the Microsoft Teams platform. In the event the focus groups will be online, we will schedule a time and date, and send you the link via the email you provide to us. With your permission, we would like to audio-record the focus group discussions.

In the first focus group, you will receive training in patient engagement and partnerships, brainstorm content for the website, and identify OI experts that should be invited to the final symposium.

During the second and third focus groups, you will review feedback received from youth with OI and their parents on the content and various features of the website. You will contribute to the design and development of the website as well. You will provide feedback on the preparation for the Symposium, and be invited to attend the event.

During the last focus group, you will be invited to finalize the website based on the findings from the Symposium, and approve any final changes allowing the research team to proceed to the next phase of study.

What will happen in this study that is "research"?

There are no experimental tests used in this study.

How long does the study last?

Each focus group will last about 1 hour, for a total of 4 hours. The final Symposium will be developed with input from the Council. This event may be for a few hours to a whole day. Overall, the study will take place over a 9-12 month period.

Can you stop being in the study?

Yes. You can drop out of the study at any time. No one will be upset. It will not affect your employment at the hospital. You can do this by telling one of the members of the research team.

There are no anticipated circumstances under which we would decide to take you off the study.

What are the risks of the study?

This study involves no more than minimal risks, which means that we expect that you will have no more risk than those you have in your normal daily life.

Are there benefits to taking part in the study?

You will not directly benefit from participating in this study. Your feedback will be used to inform the design and development of the online self-management program for adolescents with OI, which may help other adolescents to better manage their OI. If you would like to provide your contact information (email or mailing address) we will send you information on the study findings and updates on the online self- management program.

What other options are available to you?

If you decide not to enter this study, your alternative is not to be in this study.

How will information about you be kept private?

You will not be identified by name. To protect your identity, the research team will only use code numbers to refer to your information. The code numbers will be stored in a separate file. Transcriptions of the focus groups will be modified to exclude any information that may identify you. The consent forms will be stored in a locked cabinet in the PI's office at the hospital. This data will be entered into an encrypted Excel file on a password-protected computer. Any audio recordings and electronic materials will be stored on a password-protected computer in the PI’s office. Access to the filing cabinet and the computer will only be available to the research team.

If information from this study is published or presented at scientific meetings, your name and other personal information will not be used.

Your personal information may be given out if required by law. Shriners Hospital for Children Clinical Research Monitors, the McGill Institutional Review Board, employees of the funder or sponsor, or the regulator of the study may see your personal information to check on the study. By signing this consent form, you agree to let these people look at your records. We will give you a copy as well.

Data will be kept for seven years following publication of the study results. Subsequently, paper documents will be shredded and electronic data will be destroyed by secure delete according to the Retention and Destruction policy at Shriners Hospital for Children.

What are the costs?

You will not be paid for being in this study.

There are no costs to take part in this study. You will receive a personalized certificate of appreciation.

What are your rights if you take part in this study?

Voluntary Participation: Taking part in this study is your choice. You may choose not to take part in the study or you may leave at any time. Your decision about being in this study will not affect your employment at Shriners Hospitals for Children®-Canada. If you have any questions about the study, you can always talk to one of the study staff. Do not sign this form unless you have had the chance to ask questions and have received clear answers.

New Findings: We do not expect to find any new information that may affect your willingness to stay in this study during your brief involvement. However, we will tell you if there were any new findings.

Whom can you call if you have questions or problems?

If you have any questions, please ask us. If you have any questions later, please contact Dr. Argerie Tsimicalis by telephone at (514) 842-4464 ext. 2268 or by email at: atsimicalis@shrinenet.org. You can contact the Ethics Officer for the McGill IRB, Ms. Ilde Lepore, by telephone at (514) 398-8302 or by email at ilde.lepore@mcgill.ca for answers to questions you might have about research and about your rights as a research participant.

Title of Research Project: Design and Development of an E-health Program for Youth with Osteogenesis Imperfecta (Phase I)

Your signature below will show that you acknowledge and agree that:

- **You have read this consent form (or it has been read to me)**
- **You will freely take part in this research study,**
- **You have had a chance to ask questions and all of your questions have been answered**
- **You authorize the release of medical and research records for the purpose of this study**
- **You have read and understood the information above**
- **By signing this consent form, you have not given up any of my legal rights.**

You will be given a signed and dated copy of this informed consent form which is yours to keep.

CONSENT SIGNATURE:

____ __________

Signature of Participant Date

In the event of an online focus group, email: ____

....

Using language that is understandable and appropriate, I have discussed this project and the items listed above with the participant and/or his parent/legal guardian.

____ ______________________

Signature of the person who conducted the informed consent discussion Date

____ ______________________

____ ______________________

Print Name Study Role
