# Supplementary material for: The Design, Development, and Usability Testing of an eHealth Program for Youths With Osteogenesis Imperfecta: Protocol for a 2-Phase User-Centered Mixed Methods Study
Source: JMIR Res Protoc. 2023 Jun 23;12:e47524. doi: 10.2196/47524 (PMC10337436; doi:10.2196/47524)
Supplement: Multimedia Appendix 3 [file resprot_v12i1e47524_app3.pdf]

Semi-Structured Interview Guide for evaluating the e-Health Teens OI program prototype

READ:
We are interested in learning more about your online preferences and evaluating the preliminary design created for the Teens OI website (low-fi prototype). We are going to start off with some general questions about how you use internet and will then move into some more specific questions about the e-health program.

1. **We would like to know about your online preferences. For each element from the list below, could you please let me know how helpful, how frustrating, or how fun they are?**
    - Sharing pictures or stories
    - Message boards
    - Gamification (an educational approach that seeks to motivate learners by using video game design and game elements in learning environments)
    - Advertisement from OI community
    - Videos
    - Quizzes
    - Form for providing feedback
    - Save content (interesting content or to read later, such as Instagram)

Interviewer's probes
- What do you find most interesting about these items?
- How often do you use them?

2. We would like your input on evaluating the Teens OI prototype. We are going to ask for your feedback about the features and elements already created and to have new ideas.

We will present our low-fi wireframe prototype using the link from Figma: https://www.figma.com/proto/UocD1wB4c9haKrWxgPLw7o/Teens_OI_wireframe-V2?node-id=4%3A2&starting-point-node-id=4%3A2

We will share our screen and navigate through the frames (webpages prototype).

Interviewer's probes about the prototype
- What do you like about the website and why?
- What don't you like about the site and why? (Or what features are missing?)
- What features/functionality of the site do you want to have on the teens OI website?
- How can the website help you? What roles can it fulfill?
- If you had a magic wand, what would you change about this website?
- How would you name other website users who are not youth, parents, and/or healthcare professionals?
- We are interested in including resources from the OI community outside the medical field. These resources would include things about everyday life with OI. Could you share some suggestions on which themes you think would be important to include? Do you know some existing resources that you would suggest?

3. We would like to explore some other websites and get your feedback. Can you name websites that you consider good and useful (besides social media)?

Interviewer's probes

- Could you tell us one or more websites that you really like and use on the Internet? It can be for study, for entertainment, for fun, it doesn't matter. Please do not include social media.
- What do you like about the website and why?
- What don't you like about the site and why?
- What features/functionality/colors/fonts or elements of design would be interesting to have on the teens OI website?

We will explore some pre-selected websites together. When looking at the websites, we would like to know your opinion about the functionality, features, and layout of elements of the web pages.

We will share our screen and/or share the link for the following websites:

- https://www.howstuffworks.com/
- https://www.khanacademy.org
- https://teens.aboutkidshealth.ca/jiateenhub

Interview's probes about each website

- What do you like about the website and why?
- What don't you like about the site and why?
- What features/functionality/colors/fonts or elements of design would be interesting to have on teens OI website?

4. What do you think about presenting the color palettes our graphic designer suggested for the website?

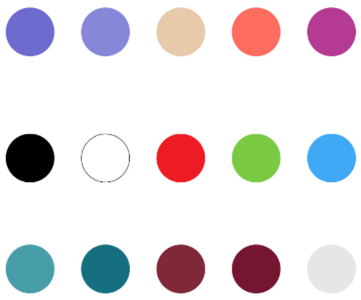

A

B

C

Interviewer's probes

- Can you tell me more about why do like them or not?

Thank you for your participation. Your feedback is important to us.

Parent - Sociodemographic questionnaire (Phase II)

Survey Flow

**Block: Default Question Block (7 Questions)**
**Branch: New Branch**<br>**If**<br>**If What is your current employment status? Please all that apply. Full time Is Selected**<br>**Or What is your current employment status? Please all that apply. Part time Is Selected**
**Standard: Working (1 Question)**
**Standard: Block 2 (13 Questions)**<br>**Standard: Block 4 (0 Questions)**

Page Break

Start of Block: Default Question Block

Q1 **Thank you for taking the time to complete this questionnaire! Please answer the following questions to the best of your ability. All your responses will be kept confidential.**

Q2 What is your relationship to the child?

- ○ Mother (biological) (1)
- ○ Father (biological) (2)
- ○ Stepmother (3)
- ○ Stepfather (4)
- ○ Guardian (5)
- ○ Other (please specify): (6) ____

Q3 How old are you (in years)?

____

Q4 What is your marital status?

- ○ Married or living common-law (1)
- ○ Single (never been married) (2)
- ○ Widow or widower (3)
- ○ Separated or divorced (4)

Page Break

Q5 Which of the following best represents your racial or ethnic heritage? Choose all that apply.

- ☐ White (1)
- ☐ South Asian (e.g., East Indian, Pakistani, Sri Lankan) (2)
- ☐ Chinese (3)
- ☐ Black (4)
- ☐ Filipino (5)
- ☐ Arab (6)
- ☐ Latin American (7)
- ☐ Southeast Asian (e.g., Vietnamese, Cambodian, Laotian, Thai) (8)
- ☐ West Asian (e.g., Iranian, Afghan) (9)
- ☐ Korean (10)
- ☐ Japanese (11)
- ☐ Other, specify: (12) ____
- ☐ Prefer not to say (13)

Q6 What is the highest level of education you have completed?

- ○ Elementary school (some or completed) (1)
- ○ Some secondary/high school (2)
- ○ Completed secondary/high school (3)
- ○ Some post-secondary (university or college) (4)
- ○ Completed university or college degree/diploma (5)
- ○ Postgraduate (6)

Q7 What is your current employment status? Please all that apply.

- ▢ Full time (1)
- ▢ Part time (2)
- ▢ Student (3)
- ▢ Unemployed (4)
- ▢ Other (please specify): (5) ____

End of Block: Default Question Block

Start of Block: Working

Q8 How many **hours a week** do you work?

____

End of Block: Working

Start of Block: Block 2

Q9 Using the scale below, how would you describe your child's health now?

	Poor (1)	Fair (2)	Good (3)	Very good (4)	Excellent (5)
(1)	○	○	○	○	○

Page Break

Q10 Do you have Osteogenesis Imperfecta (OI)?

○ No (1)

○ Yes (2)

Skip To: Q14 If Do you have Osteogenesis Imperfecta (OI)? = No

Q11 What type of OI are you diagnosed with?

○ I (1)

○ II (2)

○ III (3)

○ IV (4)

○ V (5)

○ VI (6)

○ VII (7)

○ Don't know (8)

Q12 At what age were you diagnosed with OI?

____

Q13 Using the scale below, how would you describe **your** overall health now?

	Poor (1)	Fair (2)	Good (3)	Very good (4)	Excellent (5)
(1)	○	○	○	○	○

Page Break

Q14 What type of mobile device do you use?

- ○ Cell phone only (1)
- ○ Android with data (2)
- ○ Android without data (3)
- ○ iPhone with data (4)
- ○ iPhone without data (5)
- ○ Other (please specify): (6) ____
- ○ I do not have a mobile device (7)

Q15 Do you have Internet access at home?

- ○ No (1)
- ○ Yes (2)

Q16 Using the scale below, how would you rate your comfort using the Internet?

	Very uncomfortable (1)	Somewhat uncomfortable (2)	Neutral (3)	Somewhat comfortable (4)	Very comfortable (5)
(1)	○	○	○	○	○

Q17 How much time a day do you spend on the Internet?

- ○ Do not use the Internet (1)
- ○ No time (2)
- ○ most days (3)
- ○ Less than 1 hour/day (4)
- ○ Between 1 and 4 hours/day (5)
- ○ More than 4 hours/day (6)

Page Break

Q18 What sources of support do you use on the internet? Please check all that apply.

- ▢ YouTube videos, please specify: (1) ____
- ▢ OI Facebook groups, please specify: (2) ____
- ▢ OI Foundation website (3)
- ▢ OI Federation Europe website (4)
- ▢ Shriners Hospitals for Children website (5)
- ▢ Care4BrittleBones website (6)
- ▢ Other: (7) ____
- ▢ I do not know of any sources (8)

Q19 What other sources of support do you use? Please check all that apply.

- ☐ Peers/other people with OI (1)
- ☐ Friends (2)
- ☐ Family (3)
- ☐ Other patients (4)
- ☐ Support groups (please specify): (5) ____
- ☐ Books (please specify): (6) ____
- ☐ Pamphlets (please specify): (7) ____
- ☐ Information from the hospital (8)
- ☐ Healthcare professional (please specify which type): (9) ____
- ☐ Other (please specify): (10) ____

Page Break

Q20 What is your total household income?

- ○ 0-30,000$ (1)
- ○ 30,000$-49,999$ (2)
- ○ 50,000$-74,999$ (3)
- ○ 75,000$-99,999$ (4)
- ○ 100,000$-149,999$ (5)
- ○ Over 150,000$ (6)
- ○ Prefer not to answer (7)

Q21 What type of medical coverage do you currently have?

- ▢ Provincial (1)
- ▢ State (2)
- ▢ Private (3)
- ▢ Other (please specify): (4) ____

End of Block: Block 2

Start of Block: Block 4

Semi-Structured Guide for Teens OI Usability Testing

Semi-structured interviews with youth/parent dyads and focus groups with Council members will be conducted to assess the usability of Teens OI at the SHC-Canada. The interviews and focus groups will be audio-recorded with consent from the participants. They will be conducted and facilitated by the research coordinator and/or graduate students in French and/or English, as preferred by the participants.

Below is a semi-structured guide that will be used for the youth/parent dyad interviews and the Council focus groups. The guide was adapted from a study conducted by Stinson et al. (2017), where they assessed the usability of the online self-management program, Teens Taking Charge: Managing Arthritis (Stinson et al., 2017). The guide will gather information on the usability of the Teens OI website, with a focus on its ease of use, efficiency, errors, and user satisfaction, including perceived ease of use and perceived usefulness.

The research assistant will be taking field notes throughout the focus group discussions and then sharing them with the Council in a report. This will serve as a guide for the changes to be made to the Teens OI Website. The changes will be reviewed during the second cycle of focus groups. Final changes will be addressed, and then the website will be finalized. The expected length of each focus group is 60 minutes and they will occur at a time agreed upon by members of the Council.

Now that you have used the Teens OI website, we would like to gather your feedback to help improve the usability of the website. Further, we would like to know what you liked and disliked about the Teens OI website.

Questions:

1. What did you like best about the Teens OI website?
    a. content
    b. images
    c. videos
    d. layout or tabs
    e. buttons
    f. Can you tell me more about that?
2. What did you like least about the Teens OI Website?
    a. content
    b. images
    c. videos
    d. layout or tabs
    e. buttons
    f. Can you tell me more about that?
3. Can you tell me how easy it was to navigate the website or find your way around the website?
    a. Were there any elements that made it easier to navigate the website, such as specific buttons, tabs, or images?
    b. Did the layout of the content help you find your way around the website?
    c. What are some of the challenges you encountered when navigating the website?
    d. Is there specific content or page you found hard to find?

    e. What could we add to make it easier to navigate the website and find content on the website?
4. Can you tell me what you thought of the overall look of the website?
    a. What did you think of the colours?
    b. What did you think of the images or videos?
    c. Does the website feel welcoming? Or does it look too serious?
    d. What could help make the website more visually appealing and inviting?
5. Can you tell me what you thought about the information provided on the website?
    a. Do you think the information was accurate?
    b. Do you trust the information presented on the website?
    c. Do you think the images and videos complement the information?
    d. Do you think there's enough information? Too much?
    e. Was there any missing information that should be included on the website?
    f. Was the information easy to read and understand?
    g. Do you think the information provided is useful and helpful in the self-management of OI?
6. Can you tell me whether or not you would use such a website to learn how to better manage OI?
    a. What would motivate you to use the website?
    b. What could help motivate other teens (parents) to use the website?
7. Can you tell me whether or not you think other teens (parents) would be interested in using the Teens OI website to leans more about OI self-management?
    a. What would interest them in using the website?
    b. Would you recommend this website to someone else with OI?
    c. Can you tell me more about that?
8. If you could make changes to the website, what changes would you make?
    a. Would you change the layout of the website?
    b. Would you change the information on the website?
    c. Can you tell me more about that?
9. Is there anything else you would like to tell us about the website?
    a. Can you tell me more about that?

Youth - Socio-demographic questionnaire (Phase II)

Survey Flow

**Block: Default Question Block (16 Questions)**
**Branch: New Branch**<br>**If**<br>**If What is your current employment status? Please all that apply. Full time Is Selected**<br>**Or What is your current employment status? Please all that apply. Part time Is Selected**
**Standard: Working (1 Question)**
**Standard: Marital status (2 Questions)**
**Branch: New Branch**<br>**If**<br>**If Do you have any children? No Is Selected**
**Block: If no children (2 Questions)**
**Branch: New Branch**<br>**If**<br>**If Do you have any children? Yes Is Selected**
**Block: If yes children (1 Question)**

Page Break

Start of Block: Default Question Block

Q1 **Thank you for taking the time to complete this questionnaire! Please answer the following questions to the best of your ability. All your responses will be kept confidential.**

Page Break

Q2 How old are you (in years)?

____

Q3 What is your biological sex (assigned at birth)?

- ○ Male (1)
- ○ Female (2)
- ○ Intersex (3)
- ○ Prefer not to say (4)

Q4 Which of the following best represents your racial or ethnic heritage? Choose all that apply.

- ☐ White (1)
- ☐ South Asian (e.g., East Indian, Pakistani, Sri Lankan) (2)
- ☐ Chinese (3)
- ☐ Black (4)
- ☐ Filipino (5)
- ☐ Arab (6)
- ☐ Latin American (7)
- ☐ Southeast Asian (e.g., Vietnamese, Cambodian, Laotian, Thai) (8)
- ☐ West Asian (e.g., Iranian, Afghan) (9)
- ☐ Korean (10)
- ☐ Japanese (11)
- ☐ Other, specify: (12) ____
- ☐ Prefer not to say (13)

Page Break

Q5 At what age were you diagnosed with Osteogenesis Imperfecta (OI)?

____

Q6 What type of OI are you diagnosed with?

- I (1)
- II (2)
- III (3)
- IV (4)
- V (5)
- VI (6)
- VII (7)
- Don't know (8)

Q7 Using the scale below, how would you describe your overall health now?

	Poor (1)	Fair (2)	Good (3)	Very good (4)	Excellent (5)
(1)	○	○	○	○	○

Page Break

Q8 How long have you been followed at the Shriners Hospitals for Children-Canada (SHC-Canada)? Please specify the unit of time (days, weeks, months or years).

____

Q9 How many breaks (fractures) have you experienced to date?

____

Page Break

Q10 When did your last break (fracture) happen? Enter your answer using this format: Month, Year.

____

Q11 What forms of mobility aids do you currently use?

	Never (1)	Sometimes (2)	Always (3)
Wheelchair (1)	○	○	○
Walking Aid (2)	○	○	○
Walk Independently (3)	○	○	○

Page Break

Q12 What sources of support do you use on the internet? Please check all that apply.

- ☐ YouTube videos, please specify: (1) ____
- ☐ OI Facebook groups, please specify: (2) ____
- ☐ OI Foundation website (3)
- ☐ OI Federation Europe website (4)
- ☐ Shriners Hospitals for Children website (5)
- ☐ Care4BrittleBones website (6)
- ☐ Other: (7) ____
- ☐ I do not know of any sources (8)

Q13 What other sources of support do you use? Please check all that apply.

- ▢ Peers/other people with OI (1)
- ▢ Friends (2)
- ▢ Family (3)
- ▢ Other patients (4)
- ▢ Support groups (please specify): (5) ____
- ▢ Books (please specify): (6) ____
- ▢ Pamphlets (please specify): (7) ____
- ▢ Information from the hospital (8)
- ▢ Healthcare professional (please specify which type): (9) ____
- ▢ Other (please specify): (10) ____

Page Break

Q14 What type of mobile device do you use?

- ◯ Cell phone only (1)
- ◯ Android with data (2)
- ◯ Android without data (3)
- ◯ iPhone with data (4)
- ◯ iPhone without data (5)
- ◯ Other (please specify): (6) ____
- ◯ I do not have a mobile device (7)

Q15 What is the highest level of education you have completed?

- ◯ Did not complete elementary school (1)
- ◯ Elementary School (2)
- ◯ High School (3)
- ◯ CEGEP/College (4)
- ◯ Undergraduate Degree (5)
- ◯ Graduate Degree (6)
- ◯ Other (please specify): (7) ____

Q16 What is your current employment status? Please all that apply.

- ☐ Full time (1)
- ☐ Part time (2)
- ☐ Student (3)
- ☐ Unemployed (4)
- ☐ Other (please specify): (5) ____

End of Block: Default Question Block

Start of Block: Working

Q17 How many **hours a week** do you work?

____

End of Block: Working

Start of Block: Marital status

Q18 What is your relationship status?

- ○ Single (1)
- ○ In a relationship (2)
- ○ Engaged (3)
- ○ Married (4)
- ○ Other (please specify): (5) ____

Q19 Do you have any children?

○ No (1)

○ Yes (2)

End of Block: Marital status

Start of Block: If no children

Q20 Have you considered having children?

○ No (1)

○ Yes (2)

Q21 In how many years do you think you would start having children?

○ Number of years: (1) ____

○ Don't know (2)

End of Block: If no children

Start of Block: If yes children

Q22 How many children do you have?

____

End of Block: If yes children
