# Supplementary material for: The Design, Development, and Usability Testing of an eHealth Program for Youths With Osteogenesis Imperfecta: Protocol for a 2-Phase User-Centered Mixed Methods Study
Source: JMIR Res Protoc. 2023 Jun 23;12:e47524. doi: 10.2196/47524 (PMC10337436; doi:10.2196/47524)
Supplement: Multimedia Appendix 4 [file resprot_v12i1e47524_app4.pdf]

SHC Mission Statement: Provide the highest quality care to children with neuromusculoskeletal conditions, burn injuries and other special healthcare needs within a compassionate, family-centered and collaborative care environment; Provide for the education of physicians and other healthcare professionals; and Conduct research to discover new knowledge that improves the quality of care and quality of life of children and families. This mission is carried out without regard to race, color, creed, sex or sect, disability, national origin or ability of a patient or family to pay.

2020-2021 APPLICATION CYCLE - DEVELOPMENTAL GRANT APPLICATIONS

REVIEWER CRITIQUE TEMPLATE

The SHC developmental grant mechanism supports research to be completed within 2 years and with a limited budget. SHC also accepts SHC multicenter clinical developmental applications with a maximum 3-year funding period. The primary aim of such projects is either: a) conduct a project of limited scope and duration that would not warrant an RO1 type grant application or b) serve to acquire data to be used for an R01-type application. The developmental research plan is restricted to 5 pages and may not have the same level of detail as an R01-type application. References are limited to 3 pages and may be used to demonstrate experience or working knowledge of an area or technique. Preliminary data is not required. Reviewers should evaluate the conceptual framework and experimental approach of the proposed research and its likelihood of success, major impact on the field involved, and generation of data for a new R01-type proposal.

TITLE: Development and Testing of an E-Health Program for Youth with Osteogenesis Imperfecta

INVESTIGATOR: Argerie Tsimicalis

Enter an overall impact/priority score directly in proposalCENTRAL to reflect likelihood for the developmental project to exert a sustained influence on the research fields involved, in consideration of the review criteria and additional review criteria, as applicable for the project proposed.

Scoring System utilizes a 9-point rating scale*

IMPACT	SCORE	DESCRIPTION	STRENGTHS/WEAKNESSES
High	1	Exceptional	Exceptionally strong with essentially no weaknesses
	2	Outstanding	Extremely strong with negligible weaknesses
	3	Excellent	Very strong with some minor weaknesses
Medium	4	Very Good	Strong but with numerous minor weaknesses
	5	Good	Strong but with at least one moderate weakness
	6	Satisfactory	Some strengths but also some moderate weaknesses
Low	7	Fair	Some strengths but with at least one major weakness

	8	Marginal	A few strengths and a few major weaknesses
	9	Poor	Very few strengths and numerous major weaknesses
Minor Weakness: An easily addressable weakness that does not substantially lessen impact			
Moderate Weakness: A weakness that lessens impact			
Major Weakness: A weakness that severely limits impact			

Priority to be given to applications that are responsive to the objectives of the SHC research strategic plan: "To align research with our clinical strengths and to raise SHC's international clinical research profile and reputation." Current areas of particular interest are regenerative medicine, genomics/precision medicine and motion analysis.

SCORED REVIEW CRITERIA / OVERALL SUMMARY AND RECOMMENDATIONS

Provide a separate score for each

1. Significance/Relevance (Score = 2)

Strengths:

- It is important to establish an e-Health program to improve self-care and transitional care process for youth with osteogenesis imperfecta (OI).
- This prosed project will establish this e-Health program (Teens OI) through a modification of relevant programs that have been developed for other pediatric conditions.

Weakness:

- Although the Teens OI program seems useful, when and how this program will be used are not well justified in the proposal. This is the issue related to implementation. For example, it is unclear when can teens start to use this program, how often and for how long? Does this program build a mechanism that allows teens and caregivers to communicate with clinicians? Without knowing these potential implementation options and the impact, the significance of this program is limited.

2. Investigator(s) (Score = 1)

Strengths:

- Dr. Argerie Tsimicalis (PI) has assembled a multi-disciplinary team for this proposed study.
- Team members provide complementary expertise that is required for this project.

Weakness:

- No specific concerns

3. Innovation REQUIRED (Score = 2)

Strengths:

- Many this e-Health programs have been created for teens. But very few focuses on the topics of transitional care (i.e., transiting from pediatric care to adult care).

Weakness:

- The specific content of this e-Health program to deal with transitional care issues is not clear.

4. Approach REQUIRED (Score = 4)

Strengths:

- Garrett's Theory of User Experience consisting of 5 planes of a website will be used to develop this e-Health program.
- Patient-centered and stakeholder-engagement methods will be used to assure the quality and usefulness of this e-Health program.
- The use of on-line collaborative design will be used due to COVID-19.

Weakness:

- English and French versions will be created. However, it is unclear whether the equal numbers of study participants (e.g., teens, parents and care professionals for the Council activity) will be recruited for developing English and French versions, respectively.
- 8-12 people (teens, parents and care professionals) will be invited to participate in the Council activities. If English and French versions are considered separately, each language-specific Council will contain 4-6 people. It is totally unclear how these Council members will collaborate each other (e.g., who will lead the Council meeting and how to reach the consensus?).
- It is unclear what is meant by mixed methods (basically, qualitative and quantitative approaches) to address Aim 2. If quantitative methods will be used, it is important to describe what survey tool will be conducted and how the data will be analyzed.

5. Environment REQUIRED

Strengths:

- Environment is acceptable.

Weakness:

- No specific concerns.

6. Budget and Period of Support REQUIRED

Comments:

- No specific concerns.

ADDITIONAL REVIEW CRITERIA, AS APPLICABLE *To be considered in determination of scientific merit and the priority score*

Protection for Human Subjects REQUIRED [clinical applications only]

Comments:

- No specific concerns.

Vertebrate Animals

Comments:

- Not applicable.

Biohazards

Comments:

- Not applicable.

Database/Registry

Comments:

- Not applicable.

[Revision Applications Only] ***Prior critiques available in proposalCENTRAL: click on "Resubmision" link in column to left of Applicant name***

Comments:

- The research team has carefully addressed the comments raised by reviewers, especially providing an evaluation framework and provide rationale. However, it seems the current content centers much on the design. The implementation issues (i.e., how this e-Health program can specifically help patients, parents, and clinicians to address transitional care issue) were not descried.

[Competitive Renewal Applications Only]

Comments:

Shriners Hospitals for Children®

SHC Mission Statement: Provide the highest quality care to children with neuromusculoskeletal conditions, burn injuries and other special healthcare needs within a compassionate, family-centered and collaborative care environment; Provide for the education of physicians and other healthcare professionals; and Conduct research to discover new knowledge that improves the quality of care and quality of life of children and families. This mission is carried out without regard to race, color, creed, sex or sect, disability, national origin or ability of a patient or family to pay.

2020-2021 APPLICATION CYCLE - DEVELOPMENTAL GRANT APPLICATIONS

REVIEWER CRITIQUE TEMPLATE

The SHC developmental grant mechanism supports research to be completed within 2 years and with a limited budget. SHC also accepts SHC multicenter clinical developmental applications with a maximum 3-year funding period. The primary aim of such projects is either: a) conduct a project of limited scope and duration that would not warrant an RO1 type grant application or b) serve to acquire data to be used for an R01-type application. The developmental research plan is restricted to 5 pages and may not have the same level of detail as an R01-type application. References are limited to 3 pages and may be used to demonstrate experience or working knowledge of an area or technique. Preliminary data is not required. Reviewers should evaluate the conceptual framework and experimental approach of the proposed research and its likelihood of success, major impact on the field involved, and generation of data for a new R01-type proposal.

TITLE: Development and Testing of an E-Health Program for Youth with Osteogenesis Imperfecta

INVESTIGATOR: Tsimicalis, Argerie

Enter an overall impact/priority score directly in proposalCENTRAL to reflect likelihood for the developmental project to exert a sustained influence on the research fields involved, in consideration of the review criteria and additional review criteria, as applicable for the project proposed.

Scoring System utilizes a 9-point rating scale*

IMPACT	SCORE	DESCRIPTION	STRENGTHS/WEAKNESSES
High	1	Exceptional	Exceptionally strong with essentially no weaknesses
	2	Outstanding	Extremely strong with negligible weaknesses
	3	Excellent	Very strong with some minor weaknesses
Medium	4	Very Good	Strong but with numerous minor weaknesses
	5	Good	Strong but with at least one moderate weakness
	6	Satisfactory	Some strengths but also some moderate weaknesses
Low	7	Fair	Some strengths but with at least one major weakness
	8	Marginal	A few strengths and a few major weaknesses

	9	Poor	Very few strengths and numerous major weaknesses
Minor Weakness: An easily addressable weakness that does not substantially lessen impact			
Moderate Weakness: A weakness that lessens impact			
Major Weakness: A weakness that severely limits impact			

Priority to be given to applications that are responsive to the objectives of the SHC research strategic plan: "To align research with our clinical strengths and to raise SHC's international clinical research profile and reputation." Current areas of particular interest are regenerative medicine, genomics/precision medicine and motion analysis.

SCORED REVIEW CRITERIA / OVERALL SUMMARY AND RECOMMENDATIONS

Provide a separate score for each

1. Significance/Relevance REQUIRED

Strengths: The goal of this 2-year development grant is to design, develop and test the usability of an innovative e-health program. This study will include 8-12 council members, and around 20 youth / parent dyads. The program will employ and collect sociodemographic and usability metrics, interview data, observations, field notes, and transcribed data. This data will be summarized.

This program will be measured for evidence based efficacy. The approach is strong in this evidence based approach, measuring problems, alternative strategies, benchmarks, feasibility, and hazards. The data management, PHI concerns and patient protection, and data management have been appropriately and thoroughly vetted. This is a model for this type of research should be done.

It is likely that not only will this project impact the ongoing treatment of OI, but, given the information obtained, that several high impact journal articles will result.

Weakness:

The sample size is small, (n is listed as 20 and 32 dyads in different places), and, it is not clear what can / will come out of the analysis portion of all this qualitative data, and, obviously this work will not be confirmatory, but, properly defined as pilot, feasibility, and information gathering.

2. Investigator(s) REQUIRED

Strengths: This is investigator, 10 years post-doctorate, with a developing research program and an impressive start. She is supported by Dr. Rauch, an expert in bone disease and the metabolomics of bone disease, Dr. Palomo de Oliveira a clinician with expertise in OI, Dr. Stinson who will work on data capture, Dr Veilleux, an expert in kinesiology and gait analysis, Dr. Dahan-Oliel , with expertise in transition to adult services for youth with osteogenesis imperfect provide patient engagement and mixed-methods expertise, and Dr. Hamdy, a much renowned expert in bone. It is a strong multidisciplinary team that has worked together for 7 years.

There several programmatic objectives – (1) to provide care for young OI patients, (2) provide clinician education, and (3) conduct programmatic research on the quality of life for patients and parents.

Weakness:

None noted.

3. Innovation REQUIRED

Strengths: The use of a model to develop the information, and the thorough examination of the process and information gleaned from the process is a strength. Using experience of the group, a model assessing and developing the component streams of information, there is a likelihood that a usable tool will be developed.

Weakness:

4. Approach REQUIRED

Strengths: See above. The method employed is a direct offshoot of the methods employed in the study of other diseases of muscle and bone in children by this group. It is thoughtful, iterative in that each decision point is assessed, and, if necessary, adapted/revised, and properly evaluated through out.

Weakness:

5. Environment REQUIRED

Strengths: Shriner Canada, has the largest cohort of OI subjects (n=400), and this is the right study team to tackle the problem.

Weakness:

6. Budget and Period of Support REQUIRED

Comments:

No issues.

ADDITIONAL REVIEW CRITERIA, AS APPLICABLE *To be considered in determination of scientific merit and the priority score*

Protection for Human Subjects REQUIRED [clinical applications only]

Comments: appropriate.

Vertebrate Animals

Comments:

Biohazards

Comments:

Database/Registry

Comments:

[Revision Applications Only] ***Prior critiques available in proposalCENTRAL: click on "Resubmision" link in column to left of Applicant name***

Comments: very responsive to prior review. They have moved their pilot from confirmatory to proper analytics – feasibility, acceptance, fidelity…

[Competitive Renewal Applications Only]

Comments:

Shriners Hospitals for Children™
Shriners Hospitals for Children™

CRITIQUE – RFP-2019 DEVELOPMENTAL GRANTS APPLICATIONS

TITLE: Development and Testing of an E-Health Program for Youth with Osteogenesis Imperfecta

INVESTIGATOR: Argerie Tsimicalis, PhD

***The Scoring System utilizes a 9-point rating scale (1 = exceptional; 9=poor)**

REVIEWER OVERALL SUMMARY AND RECOMMENDATIONS:

The proposal aims are to develop an English and French version of an on-line program to promote self-management of teens with osteogenesis imperfect (OI) in transition from pediatric to adult oriented health care and to test the user performance and satisfaction of the program.

The project is a second resubmission with modifications to the previous proposals including reducing the scope of the project, adding expertise to the research team that includes self-advocates and using symposium workgroups for verification of the educational modules. I remain in favor of funding the project, but not with the current budget.

The proposal remains excessively long and tedious to review. While changes have been made I am not recommending funding of this project as a priority of the applications for the current round of applicants for the Developmental Grants.

Significance REQUIRED

Is the proposal relevant to the SHC mission and does it address the importance of the problem or critical barrier to progress in the motion analysis field? If the aims are achieved, how will scientific knowledge, technical capability and/or clinical practice in the field of motion analysis be improved? How will completion of aims change the concepts, methods, technologies, treatments, services or preventative interventions that drive this field?

Strengths:
The proposal utilizes a process for program development that has been shown to be successful for children with other chronic health conditions and expanding it to the OI transition population. The product would be unique to the population and developed on a bilingual platform of both English and French.

Weakness: While a similar tool is not available, the modules proposed in the workplan have been developed over the past two years when funding has been requested. It is not clear why these modules cannot be taken up to the web and utilized currently.

Investigator(s) REQUIRED

Are the PIs, collaborators & other researchers well suited to the project? If "early stage" investigators are submitting, do they have appropriate experience and training? If established investigators, do they have

ongoing record of accomplishments that have advanced their field(s)? If project is collaborative or multi-PI, do investigators have complementary and integrated expertise?
Is leadership approach, governance and organizational structure appropriate? Is there evidence of past collaborative interaction?

Strengths:
Dr Tsimicalis is a nurse with specialty graduate training in the area of health policy and efficacy of care research. She has participated on a number of funded projects with a solid scholarly track record for her academic rank of assistant professor at McGill University. Dr. Tsimicalis collaborator, Dr Thorstad, Director of Nursing at SHC-Canada, has a strong clinical background, but limited evidence of scholarly writing, but a long history of scholarly works and innovation in the area of pediatrics. Dr. Stinson, also a nurse clinical scientist has been added to the project team. Dr Hamdy is Chief Orthopedic Surgeon at SHC and has extensive expertise with the OI population. He is an experienced clinical research with history of funding through the Shrine. Dr Rauch also has extensive research and OI experience. Dr Stinson, senior scientist at the Research Institute at the Hospital for Sick Children in Toronto is the other collaborator on the project bringing a strong background similar programming to the application, but for the management of chronic pain. Drs Palomo de Oliveira and Mitchell Bernstein have been added as a physician team members. Louis Veilleux, a doctoral level researcher and Naemi Dahan-Oliel and occupational therapist and doctoral level researcher have also been added from the Canada SHC. An OI self-advocate is identified as a project consultant.

Weakness: It is not clear why the project requires so many collaborators.

Innovation REQUIRED

Does the application challenge and seek to shift current research or clinical practice Paradigms? Will any novel theoretical concepts, approaches or methodologies, instrumentation or Interventions be utilized? Are any refinements, improvements or new application of theoretical concepts, approaches or methodologies, instrumentation or interventions proposed?

Strengths: The proposal addresses a gap with all youth who have received pediatric oriented services which is then transition to adult-oriented healthcare and progression to as independent living and employment potential as allowed by their capabilities.

Weakness: The project is a replication of similar modules that have been developed on other topics, but would create a unique tool for the use of youth with OI.

Approach REQUIRED

Are the overall strategy, methodology and analysis well reasoned and appropriate to accomplish the project specific aims? Are potential problems, alternatives and benchmarks for success presented? If in early stages of development, will strategy establish feasibility and will risky aspects be adequately managed? If the project is a clinical trial, is it multi-site? Is (a) the power analysis and (b) projected participant enrollment sufficient to insure success? Will these studies advance the field and/or clinical care of patients?

Strengths: The project will develop a Council who will receive training in patient engagement and partnerships. The Council will solicit feedback from youth with OI and parent interviews to then sponsor symposiums and presentations for the OI community to further develop Teens OI content from it's current draft to production form. Twelve (12) interactive, multi-component modules are to be designed consisting of OI-specific education, self-management strategies, and social support. The modules will be adapted from other Teens Taking Charge programs on self-management, transition, pain, psychosocial, and quality of life.

Weakness: The proposal is excessively lengthy and in areas includes excessive repetitiveness making identification of key factors laborious for the reviewer. The sampling justification explanation is not clear.

Environment REQUIRED

Will the scientific environment in which the work will be done contribute to the probability of success? Are institutional support, equipment and other physical resources available to the investigators adequate for the project proposed? Will the project benefit from unique features of the scientific environment, subject populations or collaborative arrangements?

Strengths: The SHC – Canada has a strong, international program related to OI management and is an ideal location for development of the transition program. The team of collaborators have the necessary expertise to help assure successful completion of the project as outlined and are following a formula used successfully for other education modules.

Weakness: None identified.

Human Subjects Protection THE FOLLOWING MUST BE CONSIDERED IN DETERMINATION OF SCIENTIFIC MERIT AND THE PRIORITY SCORE

Evaluate the justification for involving human subjects and the proposed protections from research risk relating to their participation according to the following review criteria: (a) Inclusion/exclusion criteria; (b) Risk to subjects; (c) Adequacy of protection against risks; (d) Potential benefits; (e) Importance of knowledge to be gained; (f) Regulatory issues (SAEs,AEs, DSMB)

Comments: Issues related to human subject's research have been addressed for the project with approval pending.

Vertebrate Animals OPTIONAL (IF APPLICABLE, THE FOLLOWING MUST BE CONSIDERED IN DETERMINATION OF SCIENTIFIC MERIT AND THE PRIORITY SCORE)

When a project involves the use of live animals, evaluate the following: (a) proposed use of animals and species, strains, sex and numbers to be used; (b) justification for use of animals and for appropriateness of the species and number proposed; (c) adequacy of veterinary care; (d) procedures for limiting discomfort, stress, pain and injury including the use of analgesic, anesthetic, and tranquilizing drugs and/or comfortable restraining devices; (e) methods of euthanasia and reason for selection if not consistent with AVMA Guidelines on Euthanasia.

Comments: NA

Biohazards OPTIONAL (IF APPLICABLE, THE FOLLOWING MUST BE CONSIDERED IN DETERMINATION OF SCIENTIFIC MERIT AND THE PRIORITY SCORE)

Assess whether materials or procedures proposed are potentially hazardous to research personnel and/or the environment and determine if adequate protection if proposed.

Comments: NA

Database/Registry (IF APPLICABLE, THE FOLLOWING MUST BE CONSIDERED IN DETERMINATION OF SCIENTIFIC MERIT AND THE PRIORITY SCORE)

Are the following questions adequately addressed in the Specific Aims section: How long has the database/registry been in existence? Who is the owner and holds the IP? Who does or will have access to the database/registry? Where is thedata stored (institution/agency)? What novel contribution/result will be obtained? What will be the benefit to Shriners physicians throughout the system?

Comments: NA

Mujltiple PI Leadership Plan IF A MULTICENTER APLICATION, THE FOLLOWING MUST BE CONSIDERED IN DETERMINATION OF SCIENTIFIC MERIT AND THE PRIORITY SCORE)

Is the governance and organizational structure of the leadership team well detailed, including communication plans, processes for making decision on direction and procedures for resolving conflict? Are the roles and administrative, technical and scientific responsibilities well delineated for the PIs and other collaborators? Is distribution of resources to specific components delineated?

Comments: The revision of the proposal includes using a cohort of self-advocates and family support members along with expertise from other centers for project development.

Budget and Period of Support REQUIRED

Consider whether the budget and requested period of support are justified and reasonable in relation to the proposed project. Budget and Period of Support. Provide reviewer recommended budget and/or comments regarding: Personnel, Consumable Supplies, Equipment, Travel, Other Expenses, including service contracts.

Comments:

With the current demonstration of efficacy of telecommunications due to the COVID pandemic the expense of bringing a team of national and international key informants to the Canada SHC is not recommended and all expenses related to the Symposia, other than a per diem should be removed from the budget.

The marketing plan for dissemination should not be limited to presentation at professional conferences. The budget justification also reads as though study findings are being disseminated when the product of the proposal is to set up modules within a website for dissemination. It is recommended that a percentage of the Conference presentation budget be used for dissemination of information on the final product.
